# Population genomics supports clonal reproduction and multiple independent gains and losses of parasitic abilities in the most devastating nematode pest

**DOI:** 10.1111/eva.12881

**Published:** 2019-11-06

**Authors:** Georgios D. Koutsovoulos, Eder Marques, Marie‐Jeanne Arguel, Laurent Duret, Andressa C. Z. Machado, Regina M. D. G. Carneiro, Djampa K. Kozlowski, Marc Bailly‐Bechet, Philippe Castagnone‐Sereno, Erika V. S. Albuquerque, Etienne G. J. Danchin

**Affiliations:** ^1^ INRA Université Côte d'Azur CNRS ISA Sophia Antipolis France; ^2^ Embrapa Recursos Genéticos e Biotecnologia Brasília Brazil; ^3^ CNRS Université Côte d'Azur IPMC Valbonne France; ^4^ Laboratoire de Biométrie et Biologie Evolutive UMR 5558 CNRS Université Lyon 1 Université de Lyon Villeurbanne France; ^5^ Área de Proteção de Plantas Instituto Agronômico do Paraná Londrina Brazil

**Keywords:** agricultural pest, clonal evolution, host races, *Meloidogyne incognita*, parallel adaptation, population genomics

## Abstract

The root‐knot nematodes are the most devastating worms to worldwide agriculture with *Meloidogyne incognita* being the most widely distributed and damaging species. This parasitic and ecological success seems surprising given its supposed obligatory clonal reproduction. Clonal reproduction has been suspected based on cytological observations but, so far, never confirmed by population genomics data. As a species, *M. incognita* is highly polyphagous with thousands of host plants. However, different *M. incognita* isolates present distinct and overlapping patterns of host compatibilities. Historically, four “host races” had been defined as a function of ranges of compatible and incompatible plants. In this study, we used population genomics to assess whether (a) reproduction is actually clonal in this species, (b) the host races follow an underlying phylogenetic signal or, rather represent multiple independent transitions, and (c) how genome variations associate with other important biological traits such as the affected crops and geographical distribution. We sequenced the genomes of 11 *M. incognita* isolates across Brazil that covered the four host races in replicates. By aligning the genomic reads of these isolates to the *M. incognita* reference genome assembly, we identified point variations. Analysis of linkage disequilibrium and 4‐gametes test showed no evidence for recombination, corroborating the clonal reproduction of *M. incognita*. The few point variations between the isolates showed no significant association with the host races, the geographical origin of the samples, or the crop on which they have been collected. Addition of isolates from other locations around the world confirmed this lack of underlying phylogenetic signal. This suggests multiple gains and losses of parasitic abilities and adaptations to different environments account for the broad host spectrum and wide geographical distribution of *M. incognita* and thus to its high economic impact. This surprising adaptability without sex poses both evolutionary and agro‐economic challenges.

## 1 INTRODUCTION

Nematodes annually cause severe damages to the world agricultural production, and the root‐knot nematodes (RKN, genus *Meloidogyne*) are the most economically harmful species in all temperate and tropical producing areas (Moens et al., 2009; Jones et al., 2013). Curiously, the most polyphagous RKN species, able to parasitize the vast majority of flowering plants on Earth (Trudgill & Blok, 2001), are described as mitotic parthenogenetic based on cytogenetics comparisons with outcrossing relatives (Triantaphyllou, 1981, 1985). This would imply absence of meiosis and obligatory asexual reproduction. Among these mitotic parthenogenetic RKN, *Meloidogyne incognita* is the most widespread species and is present, at least, in all the countries where the lowest temperature exceeds 3°C. Greenhouses over the world also extend its geographical distribution (Sasser, Eisenback, Carter, & Triantaphyllou, 1983). *Meloidogyne incognita* is so widely distributed that it is not even included on the list of regulated pests (Singh, Hodda, & Ash, 2013). Due to its worldwide distribution and extremely large range of hosts, *M. incognita* has been deemed the most damaging species of crop pest worldwide (Trudgill & Blok, 2001).

However, it has become evident that the whole broad host range of *M. incognita*, and other major RKN species, is not present in all the individuals within the species but that different “isolates” have different and overlapping ranges of compatible hosts (Moens et al., 2009). Variations regarding host range within one given species gave rise to the concept of “host race” as soon as 1952 (Sasser, 1952). Although RKN species can be differentiated based on morphological descriptions (Eisenback & Hunt, 2009), isozyme phenotypes (Carneiro, Almeida, & Quénéhervé, 2000; Esbenshade & Triantaphyllou, 1985) and molecular analysis (Blok & Powers, 2009), this is not the case of host races within a species (Triantaphyllou, 1985). Consequently, the pattern of compatibility/incompatibility of the nematode interaction with a set of different host plants was standardized into the North Carolina Differential Host Test (NCDHT, (Hartman & Sasser, 1985)) to differentiate races within Meloidogyne spp. In *M. incognita*, all the populations originally tested reproduced on tomato, watermelon and pepper and none infected peanut, but they differed in their response to tobacco and cotton defining four distinct host races (Hartman & Sasser, 1985) (Table S1). Whether some genetic characteristics are associated with *M. incognita* races remains unknown. Indeed, no correlation between phylogeny and host races was found using RAPD and ISSR markers (Baum, 1994; Cenis, 1993; Santos et al., 2012). A different attempt to differentiate host races was also proposed based on repeated sequence sets in the mitochondrial genome (Okimoto, Chamberlin, Macfarlane, & Wolstenholme, 1991). Although the pattern of repeats allowed differentiating one isolate of race 1, one of race 2 and one of race 4; the study encompassed only one isolate per race, and thus, the segregation could be due to differences between isolates unrelated to the host‐race status itself.

Hence, no clear genetic determinant underlying the phenotypic diversity of *M. incognita* isolates in terms of host compatibility patterns has been identified so far (Castagnone‐Sereno, 2006). This lack of phylogenetic signal underlying the host races is surprising because it would suggest multiple independent gains and losses of host compatibly patterns despite clonal reproduction. Theoretically, animal clones have poorer adaptability because the efficiency of selection is impaired, advantageous alleles from different individuals cannot be combined, and deleterious mutations are predicted to progressively accumulate in an irreversible ratchet‐like mode (Glémin & Galtier, 2012; Hill & Robertson, 1966; Kondrashov, 1988; Muller, 1964).

For these reasons, the parasitic success of *M. incognita* has long been described as a surprising evolutionary paradox (Castagnone‐Sereno & Danchin, 2014). However, this apparent paradox would hold true only if this species actually reproduces without sex and meiosis while presenting substantial adaptability. So far, no whole‐genome level study conclusively supports these tenets.

A first version of the genome of *M. incognita* was initially published in 2008 (Abad et al., 2008) and resequenced at higher resolution in 2017, providing the most complete *M. incognita* reference genome available to date (Blanc‐Mathieu et al., 2017). This study showed that the genome is triploid with high average divergence between the three genome copies most likely because of hybridization events. Due to the high divergence between the homoeologous genome copies, and the supposed lack of meiosis, it was assumed that the genome was effectively haploid. The genome structure itself showed synteny breakpoints between the homoeologous regions and some of them formed tandem repeats and palindromes. These same structures were also described in the genome of the bdelloid rotifer *Adineta vaga* and considered as incompatible with meiosis (Blanc‐Mathieu et al., 2017; Flot et al., 2013). However, whether these structures represent a biological reality or genome assembly artefacts remains to be clarified. Indeed, both genomes have been assembled using the same techniques and no independent biological validation for these structures has been performed. Hence, so far no conclusive evidence at the genome level supports the absence of meiosis.

Furthermore, because the reference genome was obtained from the offspring of one single female (originally from Morelos, Mexico), no information about the genomic variability between different isolates was available. Recently, a comparative genomics analysis, including different strains of *M. incognita*, showed little variation at the protein‐coding level between strains collected across different geographical locations (Szitenberg et al., 2017), confirming previous observations with RAPD and ISSR markers (Baum, 1994; Cenis, 1993; Santos et al., 2012). However, no attempt was made to associate these variations with biological traits such as the host‐race or geographical distribution. Moreover, the whole variability between isolates, including at the noncoding level, was so far never studied.

In the present study, we used population genomics analyses to investigate (a) whether the supposed absence of meiosis is supported by the properties of genomewide single‐nucleotide variant (SNV) markers between isolates, (b) the level of variation between isolates at the whole‐genome level and (c) whether these variations follow a phylogenetic signal underlying life‐history traits such as the host compatibility patterns, the geographical distribution or the current host crop plant.

**Figure 1:**
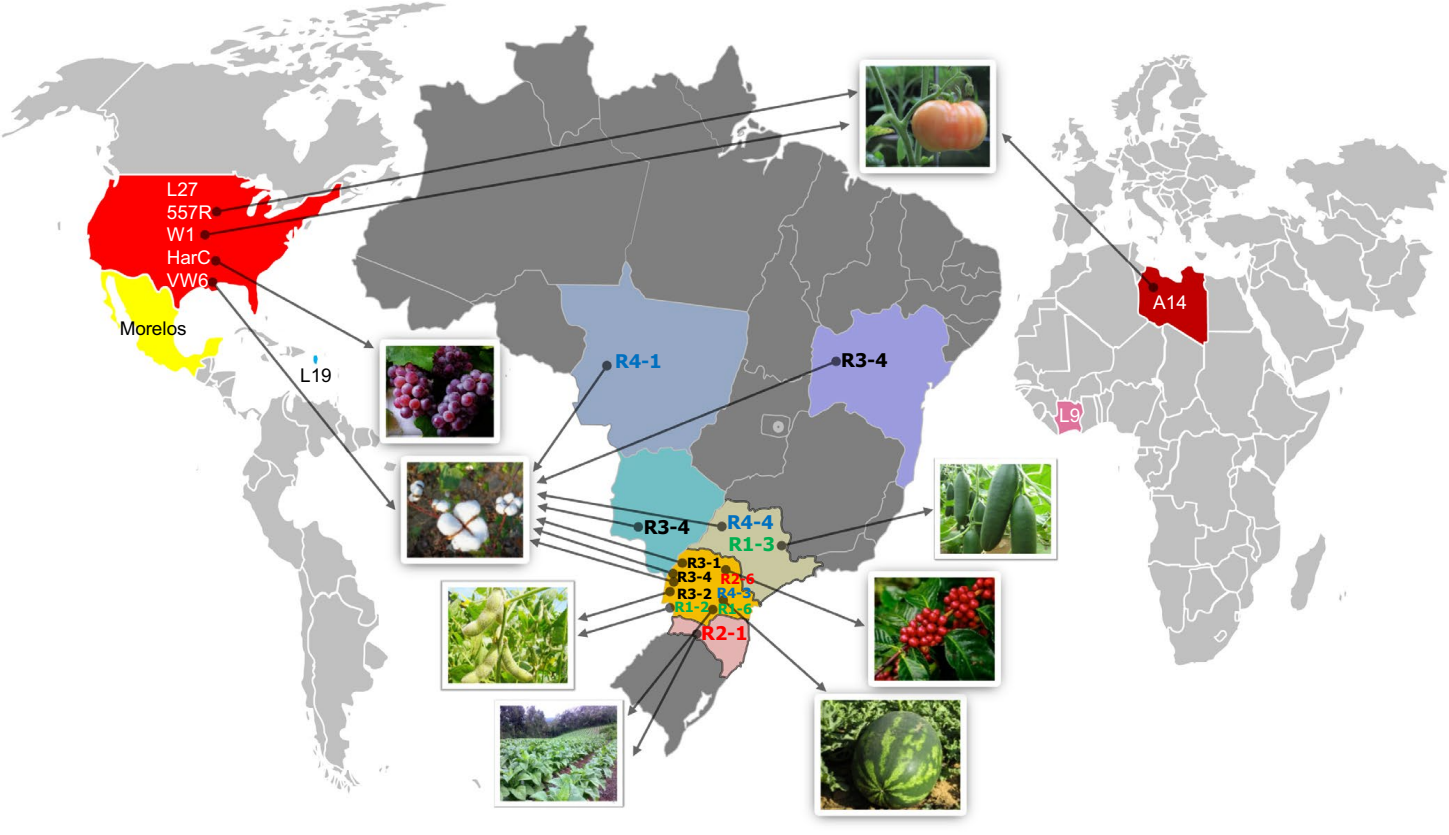
World map showing geographical origins for all samples used in the study. Expanded map of Brazil showing the states where the 11 isolates sequenced in this study were collected. Each state is highlighted with a different colour. The countries listed in the literature for other sequenced genomes are completely coloured. The crops from which the samples were isolated are illustrated by photographs, which are pointed by arrows coming from the name of the respective isolate. The names of the Brazilian isolates are in 4 different colour sources for each race (race 1 in green, 2 in red, 3 in black and 4 in blue). The names of the isolates of the literature are written in white or black

To address these questions, we have sequenced the genomes of 11 isolates covering the four *M. incognita* host races in replicates from populations parasitizing six crops across different locations in Brazil (Figure 1). We used isozyme profiles, SCAR markers and the NCDHT to characterize the biological materials and then proceeded with DNA extraction and high‐coverage genome sequencing. We identified short‐scale variations at the whole‐genome level by comparing the *M. incognita* isolates to the reference genome (Morelos strain from Mexico). We conducted several SNV‐based genetic analyses to test for evidences (or lack thereof) of recombination. Using two different approaches, we classified the *M. incognita* isolates according to their SNV patterns and investigated whether the classification was associated with the following economically important biological traits: host race, geographical localization and current host plant.

Our population genomics analysis allowed addressing key evolutionary questions such as the nature of asexual reproduction in this animal species. We also clarified the adaptive potential of this devastating plant pest in relation to its mode of reproduction. In particular, we determined whether there is a phylogenetic signal underlying variations in biological traits of agro‐economic importance such as the patterns of host compatibility (host races). While association between phylogenetic signal and patterns of host compatibilities would tend to show stable inheritance from ancestral states, the nonassociation would support multiple gains and losses of parasitic abilities and substantial adaptability.

This resolution has important agricultural and economic applications since crop rotation and other control strategies should take into account the adaptive potential of this nematode pest.

## 2 MATERIALS AND METHODS

### 2.1 Purification and characterization of *M. incognita* isolates

The *M. incognita* isolates (Table 1) originate from populations collected from different crops and geographically distant origins in Brazil (Figure 1). For each isolate, one single female and its associated egg mass were retrieved as explained in Carneiro and Almeida (2001). To determine the species (here *M. incognita*), we used esterase isozyme patterns on the female (Carneiro et al., 2000). The corresponding single egg mass was used for infection and multiplication on tomato plants (*Solanum lycopersicum* L. cv. Santa Clara) under greenhouse conditions at a temperature of 25–28°C. After 3 months, we confirmed the *M. incognita* species using esterase phenotypes (Carneiro & Almeida, 2001). Once enough nematodes were multiplied, a pool was collected and we performed the North Carolina Differential Host Test (NCDHT) (Hartman & Sasser, 1985) with the following plants: cotton cv. Deltapine 61, tobacco cv. NC95, pepper cv. Early California Wonder, watermelon cv. Charleston Gray, peanut cv. Florunner and tomato cv. Rutgers to determine the host‐race status. We inoculated these plants with 5,000 eggs and second‐stage infective juveniles (J2) of *M. incognita* and maintained them under glasshouse conditions at 25–28°C for 3 months, with watering and fertilization as needed. Two months after inoculation, the root system was rinsed with tap water, and egg masses were stained with Phloxine B (Hartman & Sasser, 1985) to count the number of galls and eggs masses separated for each root system. We assigned a rating index number according to the scale: 0 = no galls or egg masses; 1 = 1–2 galls or egg masses; 2 = 3–10 galls or egg masses; 3 = 11–30 galls or egg masses; 4 = 31–100 galls or egg masses; and 5 > 100 galls or egg masses per root system (Table 1). Host–plants types that have an average gall and egg mass index of 2 or less are designated nonhost (−). The other plants (index ≥ 3) are designated hosts (+). We categorized *M. incognita* host races based on their ability to parasitize tobacco and cotton (Table 1). Classically, the index for Rutgers tomato (susceptible control) is higher than 4 (+) (Hartman & Sasser, 1985). The rest of the population was kept for multiplication on tomato plants to produce enough nematodes for sequencing (typically >1 million individuals pooled together).

**Table 1:** Host‐race characterization of the 11 *Meloidogyne incognita* isolates used in this study

Race	ID^a^	Host Crop^b^	PID^c^	Geographical origin	Esterase phenotype	NCDHT note^d^						References
						tb	tm	wm	pt	pr	ct	
1	R1‐2	soybean	INC15	Londrina—PR	I2	0	5	4	0	5	0	Mattos et al. 2016
	R1‐3	cucumber	ND	Piracicaba—SP	I2	0	5	5	0	4	0	
	R1‐6	tobacco	LGM39	Mercedes—PR	I2/ I1	0	5	5	0	4	0	Filho, Machado, Dallagnol, & Aranha Camargo, 2016
2	R2‐1	tobacco	LGM09	Sombrio—SC	I2	5	5	4	0	4	0	Filho et al. 2016
	R2‐6	coffee	22B	São Jorge do Patrocínio—PR	I1	4	5	5	0	4	1	
3	R3‐1	cotton	PR‐3	Umuarama—PR	I2	0	5	5	0	5	5	da Silva et al. 2014
	R3‐2	soybean	ND	Londrina—PR	I2	0	5	5	0	5	5	
	R3‐4	cotton (pool)	PR‐3 Umu, PR‐3 Lon, MTS‐R3, BA‐R3	Umuarama—PR, Londrina—PR, Dourados—MS, L.E.Magalhães—BA	I2	0	5	5	0	5	5	da Silva et al. 2014
4	R4‐1	cotton	MT‐4	Campo Verde—MT	I2	5	5	5	0	5	5	da Silva et al. 2014
	R4‐3	watermelon	NG	Londrina—PR	I2	5	5	5	0	5	5	
	R4‐4	cotton	GEN 306	Vargem Grande do Sul—SP	I1	5	5	5	0	5	5	

Abbreviations: ct, cotton “Deltapine 61”; pr, pepper “Early California Wonder,”; pt, peanut “Florunner,”; tb, tobacco “NC95,”; tm, tomato “Rutgers,”; wm, watermelon “Charleston Gray,”.

a Isolate identification code.

b Host plant crop (soybean, *Glycine max*; cucumber, *Cucumis sativus*; tobacco, *Nicotiana tabacum*; coffee, *Coffea arabica*; cotton, *Gossypium hirsutum*.; watermelon, *Citrullus vugaris*.

c Population id.

d Host range results for the North Carolina Differential Host Test (NCDHT), numbers represent gall index with 0 = no galls; 1 = 1‐2; 2 = 3‐10; 3 = 11‐30; 4 = 31‐100; and 5 = more than 100 galls.

### 2.2 DNA preparation and SCAR test

For each characterized nematode isolate, we extracted and purified the genomic DNA from pooled eggs with the supplement protocol for nematodes of the QIAGEN Gentra^®^ Puregene^®^ Tissue Kit with the following modifications: incubation at 65°C in the cell lysis buffer for 30 min and incubation at 55°C with proteinase K for 4 hr. We verified DNA integrity on 0.8% agarose gel and the DNA quantification on Nanodrop. We confirmed isolate species purity by SCAR‐PCR (Randig, Bongiovanni, Carneiro, & Castagnone‐Sereno, 2002; Zijlstra, Donkers‐Venne, & Fargette, 2000) using the SCAR primers specified in Table S6 for the RKN *M. javanica, M. paranaensis, M. incognita* and *M. exigua*.

### 2.3 Sequencing library preparation

We assessed input gDNA quantity using Qubit and normalized the samples to 20 ng/μl as described in TruSeq^®^DNA PCR‐Free Library Prep Reference Guide (#FC‐121–3001, Illumina) prior fragmentation to 350 bp with Covaris S2. We assessed the quality of fragments after size selection and size control of the final libraries using High Sensitivity DNA Labchip kit on an Agilent 2100 Bioanalyzer.

### 2.4 Whole‐genome sequencing

We quantified sample libraries with KAPA library quantification kit (#7960298001, Roche) twice with two independent dilutions at 1:10,000 and 1:20,000. We calculated the average concentration of undiluted libraries and normalized them to 2 nM each then pooled them for sequencing step.

We generated high‐coverage genomic data for the 11 *M. incognita* isolates by 2 × 150 bp paired‐end Illumina NextSeq 500 sequencing with High Output Flow Cell Cartridge V2 (#15065973, Illumina) and High Output Reagent Cartridge V2 300 cycles (#15057929, Illumina) on the UCA Genomix sequencing platform, in Sophia‐Antipolis, France. We performed two runs to balance the read's representation among the isolates and obtain homogeneity of coverage for the different samples (Table S2).

### 2.5 Variant detection

We trimmed and cleaned the reads from each library with cutadapt (Martin, 2011) to remove adapter sequences and bases of a quality inferior to 20. We mapped the clean reads to the *M. incognita* reference genome (Blanc‐Mathieu et al., 2017), using the BWA‐MEM software package (Li, 2013). This reference genome is described as triploid with three equally highly diverged A, B and C genome copies as a result of hybridization events. Most of the triplicated regions have been correctly separated during genome assembly, according to genome assembly size (183.53 Mb) that is in the range of the estimated total DNA content in cells via flow cytometry (189 ± 15 Mb) (Blanc‐Mathieu et al., 2017)). Hence, the genome was considered in this analysis as mostly haploid. However, the distribution of per‐base coverage on the genome assembly presented a 2‐peaks distribution with a second minor peak at ~twice the coverage of the main peak (Figure S2). Genome regions of double coverage most likely represent cases where two of the three homoeologous loci have been collapsed during the assembly, probably due to lower divergence. Such regions will systematically be responsible for “artefactual” 0/1 SNV (presenting variations within isolates) as the reads from the two homoeologous copies will map a single collapsed region in the reference genomes. To avoid confusion between SNV representing true variations between individuals within isolates from those being artefacts due to collapsed homoeologous regions, 0/1 SNV were discarded from the analysis and only 1/1 SNV fixed within isolates were considered.

We used SAMtools (Li et al., 2009) to filter alignments with MAPQ lower than 20, sort the alignment file by reference position and remove multimapped alignments.

We used the FreeBayes variant detection tool (Garrison & Marth, 2012) to call SNV and small‐scale insertions/deletions, incorporating all the library alignment files simultaneously and produced a variant call file (VCF). We filtered the resulting VCF file with the vcffilter function of vcflib (Anon, 2018), retaining the positions that had more than 20 Phred‐scaled probability (QUAL) and a coverage depth (DP)> 10. As a comparison with an outcrossing meiotic diploid nematode, we conducted the same analyses on the genome of *Globodera pallida* (Eves‐van den Akker et al., 2016). We first phased the SNV to haplotypes using WhatsHap (Martin et al., 2016) because the genome assembly mainly consists of collapsed paternal and maternal haplotypes.

### 2.6 Genetic tests for detection of recombination

We used custom‐made scripts (cf. Data Accessibility section) to calculate the proportion of fixed markers passing the 4‐gametes test and linkage disequilibrium (LD) *r* ^2^ values as a function of intermarker distance along the *M. incognita* and *G. pallida* genome scaffolds.

### 2.7 Genetic diversity between isolates, clusters and efficacy of purifying selection

We used bpppopstats from the Bio++ libraries (Guéguen et al., 2013) to estimate the nucleotide variability at nonsynonymous and synonymous sites as well as efficacy of purifying selection (*π*_N_, *π*_S_ and *π*_N_/*π*_S_) using a multiple alignment of the coding regions. We calculated fixation index (*F*_ST_) for the three clusters using vcftools (Danecek et al., 2011).

### 2.8 Principal component analysis

We performed a principal component analysis (PCA) to classify the isolates according to their SNV patterns and mapped the race characteristics, geographical location or current host plants on this classification. We used the filtered VCF file as input in the statistical package SNPRelate (Zheng et al., 2012) to perform the PCA with default parameters.

### 2.9 Phylogenetic analysis

Based on the VCF file and the *M. incognita* gene predictions (Blanc‐Mathieu et al., 2017), we selected 85,413 coding positions that contained synonymous or nonsynonymous mutations. We aligned these positions and then used them as an input in SplitsTree4 with default parameters. The resulting network produced a bifurcating tree that was identical to the one obtained with RAxML‐NG using GTR+G+ASC_LEWIS model. The bifurcating tree was used as input to PastML (Ishikawa, Zhukova, Iwasaki, & Gascuel, 2018) for reconstruction of the ancestral states of ability to parasitize tobacco and cotton (Figure S8). Phylogenetic inferences for the largest scaffolds containing at least 20 SNV and the mitochondrial genome were conducted with RAxML‐NG (Kozlov, Darriba, Flouri, Morel, & Stamatakis, 2019) using the GTR+G substitution model (except for scaffolds 10 and 20 for which the K80+G model was used because not enough phylogenetically informative positions were available).

### 2.10 Test for association between biological traits and genetic clusters

We used Fisher's exact test in R to assess whether there was a significant association between the SNV‐based clusters and the host races or the crop species from which the isolates were originally collected. We also conducted an isolation‐by‐distance (IBD) analysis using the adegenet R package (Jombart & Ahmed, 2011) to check how well the genetic distances correlate with geographical distances between the sampling points of the isolates. Geographical distances were calculated from exact sampling locations, when available, or centre points if the region was known but not the exact sampling location. Sample R3‐4 was excluded from this analysis since it was a mix of samples pooled together from different geographical locations. L27 was also excluded since the sampling location was unknown.

### 2.11 Mitochondrial genome analysis

We subsampled genomic clean reads to 1% of the total library for each *M. incognita* isolate. Then, we assembled them independently using the plasmidSPAdes assembly pipeline (Antipov et al., 2016). We extracted the mitochondrial contigs based on similarity to the *M. incognita* reference mitochondrial genome sequence (NCBI ID: NC_024097). In all cases, the mitochondrion was assembled in one single contig. We identified the two repeated regions (63 bp repeat and 102 bp repeat), described in Okimoto et al. (1991), and we calculated the number of each repeat present in these regions.

## 3 RESULTS

### 3.1 The *M. incognita* genome is mostly haploid and shows few short‐scale variations

We collected 11 *M. incognita* populations from six different states across Brazil and from six different crops (soybean, cotton, coffee, cucumber, tobacco, watermelon) (Figure 1). Each isolate was reared by multiplication of the egg mass of one single female on tomato plants (methods). After having confirmed that the 11 isolates were actually *M. incognita*, we characterized their host‐race status using the NCDHT (methods, Table 1). We characterized three isolates as race 1, two as race 2, three as race 3, and three as race 4.

We generated paired‐end genome reads (~76 million per isolate) which covered the ~184 Mb *M. incognita* genome assembly (Blanc‐Mathieu et al., 2017) at a depth >100X (Table S2) for each isolate. Variant calling, performed in regions with at least 10x coverage per sample, identified 338,960 polymorphic positions (~0.19% of the total number of nonambiguous nucleotides). Around 20% of these positions corresponded to 1/1 SNV, fixed within each isolate but variable between at least one isolate and the reference genome. We examined the distribution of base coverage of SNV fixed within isolates (1/1 fixed SNV) and SNV that presented variations within at least one isolate (0/1 SNV). We observed that the 0/1 SNV, which were variable within isolates, showed a peak of distribution at ~ twice the coverage of the peak for fixed 1/1 SNV in the 11 isolates (Figure S1). This parallels the distribution of base coverage in the *M. incognita* reference genome scaffolds which shows a major peak at ~65X and a second minor peak at ~130X (twice the coverage; Figure S2). These genome regions at double coverage were considered as representing highly similar pairs of homoeologous genome copies that were collapsed during the assembly (Blanc‐Mathieu et al., 2017). Although these regions are minority in the genome assembly, they seem to be responsible for many 0/1 SNV (presenting within isolate variations). The SNV in these minority regions of double coverage probably results from genome reads of two homoeologous regions mapped to a single collapsed region in the reference assembly. Hence, most of these 0/1 SNV might not represent variations between individuals within an isolate but between the few collapsed homoeologous regions. Because the reference genome is mostly assembled in haploid status (Figure S2), and the nature of 0/1 SNV is ambiguous, we will utilize only 1/1 SNV fixed within isolates as markers for all downstream analyses. Although this precludes analyses of variations between individuals within isolates, this allows a comparison of variations between isolates based on >66,000 solid fixed markers.

### 3.2 No evidence for meiotic recombination in *M. incognita*

**Figure 2:**
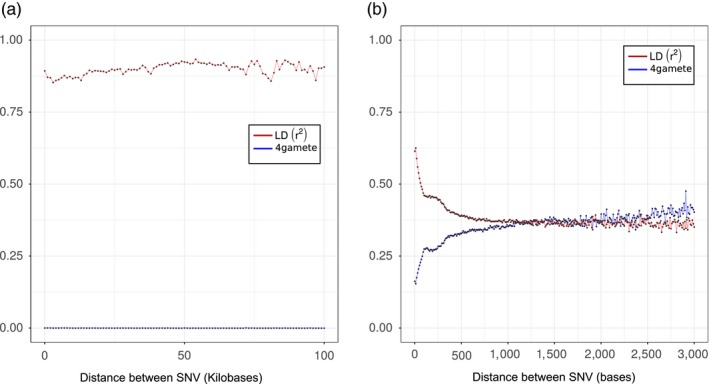
Linkage Disequilibrium and 4‐gametes test of *M. incognita* (a) and *G. rostochiensis* (b) isolates. The *r* ^2^ correlation between markers, indicating linkage disequilibrium (LD), is given as a function of the physical distance between the SNV markers (red line). The proportion of pairs of two‐state markers that pass the 4‐gamete test is given as a function of the distance between the markers (blue line). (a): on *M. incognita* scaffolds and (b): on *G. rostochiensis* phased haplotypes

Based on cytogenetics observation, *M. incognita* and other tropical root‐knot nematodes have been described as mitotic parthenogenetic species (Triantaphyllou, 1981, 1985). However, this evolutionary important claim has never been confirmed by genome‐wide analyses so far. Using the fixed SNV markers at the whole‐genome scale, we conducted linkage disequilibrium (LD) analysis as well as 4‐gametes test to search for evidence for recombination (or lack thereof). In an outcrossing species, physically close markers should be in high LD, with LD substantially decreasing as distance between the markers increases, because of recombination, and eventually reach absence of LD similarly to markers present on different chromosomes. In clonal species, however, in the absence of recombination, the LD between markers should remain high and not decrease with increasing distance between markers. By conducting an analysis of LD, we did not find any trend for a decrease of LD between markers as a function of their physical distance (Figure 2a). In contrast, the LD values remained high regardless the distance and oscillated between 0.85 and 0.94. Hence, we did not observe the expected characteristic signatures of meiosis. An inversely contrasted situation between outcrossing and clonal genomes should be observed for the 4‐gametes test. Taking fixed SNV markers that exist in two states among the 11 isolates, the proportion of pairs of markers that pass the 4‐gametes test (i.e., that represent the 4 products of meiosis) should rapidly increase with distance between the markers in case of recombination. In contrast, in the absence of recombination, no trend for an increase of the proportion of pairs or markers passing the 4‐gametes test with distance between markers should be observed. By conducting an analysis of 2‐states markers, we observed no trend for a change in the proportion of markers passing the test with distance. In contrast, the distribution remained flat and close to a value of 0.0 (Figure 2a). Again, this trend does not correspond to the expected characteristic of meiotic recombination.

To assess the sensitivity of our method in finding evidence for recombination, we conducted the same analyses (LD and 4‐gametes tests) in the outcrossing diploid meiotic plant–parasitic nematode *Globodera rostochiensis* (Eves‐van den Akker et al., 2016). Because the *G. rostochiensis* genome assembly mostly consists of merged paternal and maternal haplotypes, we had to phase the SNVs before conducting LD and 4‐gametes tests. The results were totally contrasted between *M. incognita* and *G. rostochiensis* (Figure 2b). In *G. rostochiensis*, the LD and 4‐gametes curves started at relatively lower (<0.7) and higher (>0.15) values, respectively. Furthermore, we observed a rapid exponential decrease of r^2^ in the first kb for LD. At an intermarker distance of 3 kb, the *r* ^2^ value was <0.37. In parallel, we observed a concomitant rapid and exponential increase in the proportion of markers passing the 4‐gametes test, which was >0.38 at the same intermarker distance. Hence, while *G. rostochiensis* appears to display all the expected characteristics of meiotic recombination, this was not the case for *M. incognita*. This validates at a whole‐genome scale the lack of evidence for meiosis previously proposed based on cytological observations in *M. incognita*.

### 3.3 The SNV between isolates are sparse, rarely in genes and not specific to races

**Figure 3:**
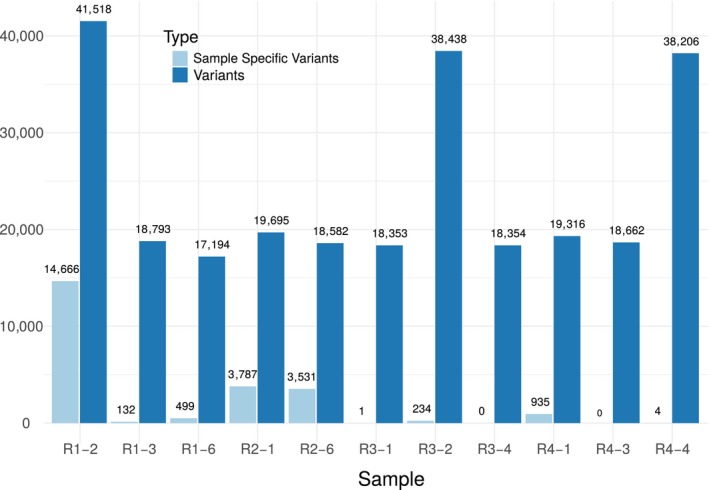
Distribution of the number of variants per race and isolate. Number of variants per isolate (dark blue) and isolate‐specific variants (light blue) for the 11 Brazilian isolates

Each isolate showed a different level of divergence from the reference genome with R1‐2 having the highest number of fixed SNV (41,518) and R1‐6 having the least (17,194) variants (Figure 3). Furthermore, even though the R3‐4 isolate originated from a pool of four populations, the low number of SNV compared to the reference indicates either that the genomes of these four populations were very close genetically or that a specific population displaced the other three (Figure 3). Thus, the R3‐4 isolate was analysed exactly as the other isolates. Overall, the fixed SNV on the nuclear genomes of the eleven isolates, compared to the Morelos reference strain, spanned between 0.01% and 0.02% of the nucleotides. In comparison, SNV in the mitochondrial genome spanned between 0.04% and 0.18% of the nucleotides.

Interestingly, race‐specific variants exist only for race 2, which exhibited 30 race‐specific variations. This is possibly due to the fact that race 2 is represented by only two isolates (vs. 3 for the rest of the isolates).

Overall, the vast majority (~78%) of SNV were outside of coding regions; only 14,704 variable positions fell in coding regions and covered 7,259 out of 43,718 predicted protein‐coding genes. In these coding regions, 8,179 were synonymous substitutions with no evident functional impact. A total of 3,854 SNV yielded nonsynonymous substitution. Interestingly, 45 of these SNV fell in 16 different effector genes (Table S3). These effector genes are known to be specifically expressed in the nematode secretory dorsal (15 SNVs on four genes) or subventral gland cells (30 SNV in 12 genes) and encode proteins secreted into plant tissue to support parasitic functions (Vieira & Gleason, 2019). Although the nonsynonymous substitutions might have an impact on plant parasitism, their occurrences did not correlate with the four host races or with a particular infected crop. We also identified 93 nonsense mutations and the rest was constituted by other disruptive mutations, none of them falling in known effector genes.

From the SNV falling in coding regions, we constructed a multiple alignment and measured nucleotide diversity at synonymous (*π*_s_) and nonsynonymous (*π*_n_) sites for the 11 isolates as well as the *π*_n_/*π*_s_ ratio as a measure of the efficiency of selection. Consistent with the overall low number of SNV, the *π*_s_ value across the 11 isolates was low (1.29 × 10^−03^). This is one order of magnitude lower than the values measured for two outcrosser nematodes from the *Caenorhabditis* genus (Romiguier et al., 2014), *C. doughertyi* (formerly sp. 10:4.93 × 10^−02^) and *C. brenneri* (3.22 × 10^−02^). A similar difference of one order of magnitude was also observed for the diversity at nonsynonymous sites with a *π*_n_ value of 1.66 × 10^−04^ for *M. incognita* and values reaching 2.53 × 10^−03^ and 1.28 × 10^−03^ for *C. doughetryi* and *C. brenneri*, respectively. However, the *π*_n_/*π*_s_ ratio was substantially higher for *M. incognita* (0.129) than for the two outcrossing Caenorhabditis (0.051 and 0.040 for *C. doughetryi* and *C. brenneri*, respectively). These results suggest a lower efficacy of selection in the obligate parthenogenetic *M. incognita* than in the two outcrossing *Caenorhabditis* nematodes.

### 3.4 There is no significant association between the short‐scale variants and the biological traits

**Figure 4:**
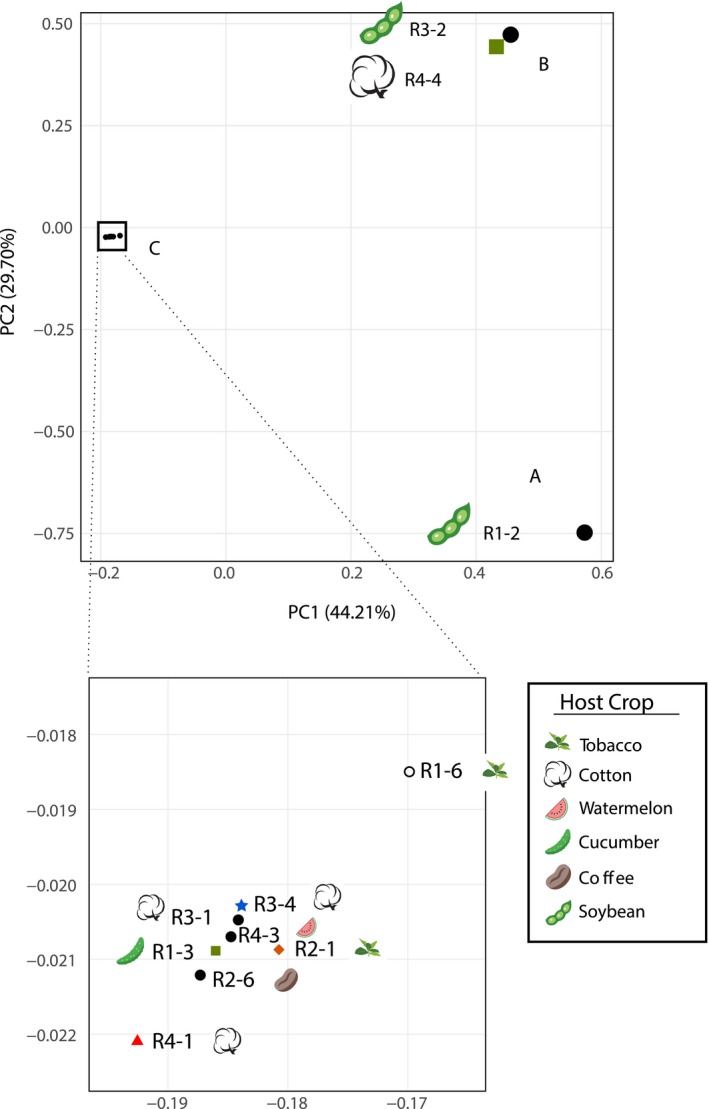
PCA of the Brazilian *M. incognita* isolates groups them into three clusters (A, B, and C). The geographical origins are associated with coloured shapes: black circle: Paraná, orange diamond: Santa Catarina, green square: São Paulo, red triangle: Mato Grosso, blue star: pool. Host plant representative pictures are displayed next to the isolates: soybean pod (R1‐2 and R3‐2); cotton flower (R3‐1, R3‐4, R4‐4, and R4‐1); coffee grain (R2‐6); cucumber vegetable (R1‐3); tobacco leaves (R1‐6 and R2‐1); and watermelon fruit slice (R4‐3)

Using principal component analysis (PCA) on the whole set of fixed SNV, we showed that the eleven *M. incognita* isolates formed three distinct clusters, which we named A, B and C (Figure 4). Cluster A is represented by isolate R1‐2 alone, which has the highest number of variants. Cluster B is constituted by R3‐2 and R4‐4. The rest of the isolates fall in a single dense cluster C of overall low variation. There was no significant association between the clusters and the host‐race status (Fisher's exact test *p*‐value = 1, Appendix S1, Table S4). This implies that isolates of the same host race are not genetically more similar to each other than isolates of different host races. There was also no significant association between the SNV‐based clusters and the crop plant from which nematodes were collected (Fisher's exact test *p*‐value = 0.69, Appendix S1, Table S4). Interestingly, the four different host races are all represented in one single cluster (C). Within this cluster, the total number of variable positions was 29,597, meaning that the whole range of host‐race diversity is present in a cluster that represents only 44% of the total existing genomic variation. We also conducted an isolation‐by‐distance (IBD) analysis, which showed no correlation between the genetic distance and the geographical distance (Figure S3).

To assess the levels of separation versus past genetic exchanges between these clusters, we calculated fixation index values (F_ST_). Weighted F_ST_ values between clusters were all >0.83, suggesting a lack of genetic connections between the clusters (Table S5). Using the mean F_ST_ values, in contrast, while we observed a mean F_ST_ > 0.98 between clusters A and B, indicating a lack of genetic connection between R1‐2 and cluster B, the F_ST_ values were much lower between A and C (0.35) and between B and C (0.52). This would suggest isolates from clusters A and B both result from a past bottlenecked dispersal and propagation from some isolates in cluster C. We also conducted the same *π*_n_/*π*_s_ analysis than the one performed at the whole species level for each cluster of the PCA containing at least 2 isolates. These cluster‐specific statistics yielded similar *π*_n_/*π*_s_ ratio than the one observed at whole species level (Cluster C: *π*_s_ 3.8 × 10^−04^, *π*_n_ 5.36 × 10^−05^, *π*_n_/*π*_s_ 0.141; Cluster B: *π*_s_ 2.08 × 10^−05^, *π*_n_ 2.64 × 10^−06^, π_n_/π_s_ 0.127).

### 3.5 Phylogenetic networks confirm the lack of association of SNV with biological traits and support clonal evolution

**Figure 5:**
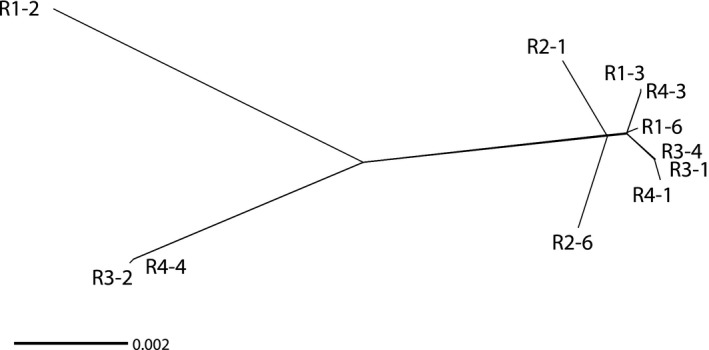
Phylogenetic network for *M. incognita* isolates based on SNV present in coding sequences. The phylogenetic network based only on changes in coding sequences produced a bifurcating tree and shows the same grouping than the PCA analysis, into 3 distinct clusters

Using a phylogenetic network analysis based on SNV present in coding regions, we could confirm the same three clusters (Figure 5). This further supports the absence of phylogenetic signal underlying the host races (patterns of host compatibilities). Interestingly, this network analysis based on fixed SNV yielded a bifurcating tree and not a network. This result further supports a lack of genetic exchanges between the isolates and clonal reproduction. To confirm this result, we conducted separate phylogenetic analyses for each of the 14 longest scaffolds with sufficient number of phylogenetically informative variable positions and the mitochondrial genome. All these analyses showed a clear separation between the same three clusters (A, B and C) with some minor polytomies within cluster C (Figure S5 and Figure S6).

According to the two classification methods (PCA and phylogenetic network), isolate R1‐2 seemed to be the most divergent from the rest of isolates, which is consistent with its higher total number of SNV and number of isolate‐specific SNV. Then, a small cluster was composed of isolates R3‐2 and R4‐3 (equivalent to cluster B of the PCA). Finally, a cluster (equivalent to PCA cluster C) grouped the rest of the eight isolates and covered all the defined host races as well as 5 of the 6 different host plants. Consistent with the PCA and phylogenetic network analysis, we also did not observe significant association between the number of repeats in the two repeat regions in the mitochondrial genome (63R and 102R) and races, geographical origin or host plant of origin (Table 2).

**Table 2:** Number of repeats per region (63 nt and 102 nt) in the mitochondrial DNA of each isolate; decimals indicate truncated repeats

ID	63 nt Region	102 nt Region	Location	Host plant
R1‐2	7.3	5.5	Londrina—PR	Soybean
R1‐3	7	13	Piracicaba—SP	Cucumber
R1‐6	1.2	7	Mercedes—PR	Tobacco
R2‐1	7	15.4	Sombrio—SC	Tobacco
R2‐6	7	9	São Jorge do Patrocínio—PR	Coffee
R3‐1	7	13	Umuarama—PR	Cotton
R3‐2	14	8.3	Londrina—PR	Soybean
R3‐4	6	13	Umuarama—PR Londrina—PR Dourados—MS L.E.Magalhães—BA	Cotton
R4‐1	6	14.7	Campo Verde—MT	Cotton
R4‐3	3	9	Londrina—PR	Watermelon
R4‐4	14	8.3	Vargem Grande do Sul—SP	Cotton

### 3.6 Addition of further geographical isolates does not increase the genomic diversity and confirms the lack of association between genetic distance and biological traits

**Figure 6:**
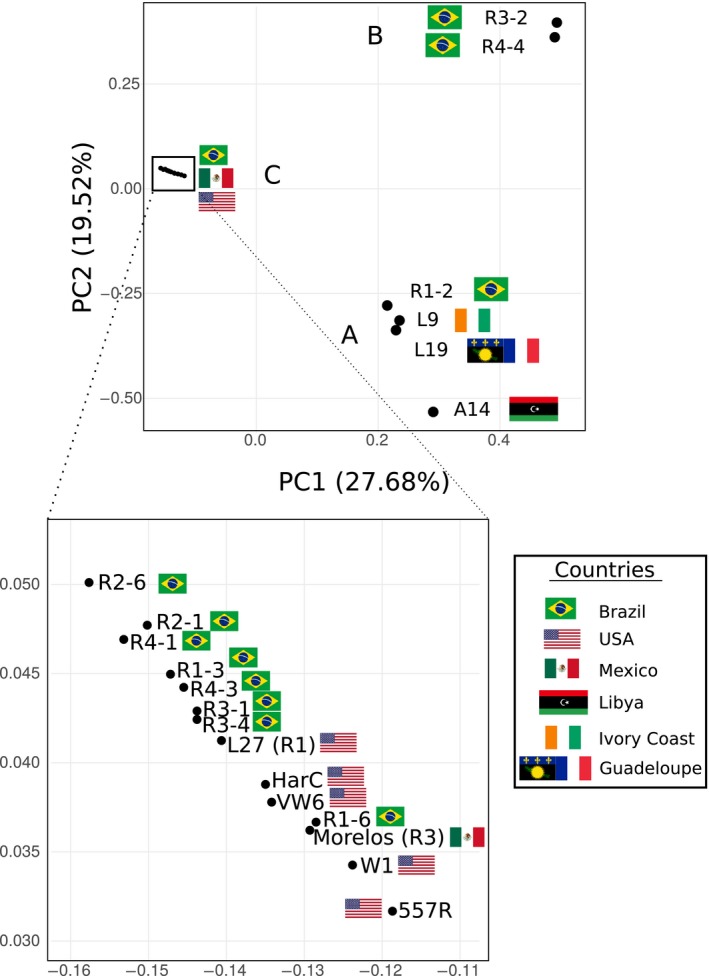
PCA of all publicly available genomes for *M. incognita* isolates, worldwide. The isolates were regrouped based on SNV patterns confirming the same three clusters. Origin countries are indicated by flags (Brazil for R1‐2, R1‐3, R1‐6, R2‐1, R2‐6, R3‐1, R3‐2, R3‐4, R4‐1, R4‐3, R4‐4; United States for L27, 557R, HarC, W1, VW6; Mexico for Morelos; Libya for A14; Ivory Coast for L9; Guadeloupe Island in the French Antilles for L19)

To investigate more widely the diversity of *M. incognita* isolates in relation to their mode of reproduction and other biological traits, we included whole‐genome sequencing data from additional geographical isolates (Szitenberg et al., 2017). These genome data included one isolate from Ivory Coast, one from Libya, one from Guadeloupe (French West Indies) and five from the United States (Figure 1). We pooled these eight new isolates with the eleven Brazilian isolates generated in this study as well as the *M. incognita* Morelos strain (reference genome) and performed a new PCA with the same methodology. Astonishingly, adding these new isolates recovered the same separation in three distinct clusters (A, B and C) (Figure 6). All the new isolates from additional and more diverse geographical origins fell in just two of the previous Brazilian clusters (A and C). Cluster A that previously contained R2‐1 alone, now encompasses the Ivory Coast, Libyan and Guadeloupe isolates. Cluster C that previously contained eight of the Brazilian isolates and covered all the host races now includes the five US isolates as well as the Mexican isolate (Morelos, reference genome). Cluster B remains so far Brazilian‐specific with only R3‐2 and R4‐4 in this cluster. Addition of the new geographical isolates did not substantially increase the number of detected variable positions in the genome. Analyses ran with this whole set of available *M. incognita* isolates also further supported the lack of association of SNV‐based clusters with biological traits such as host races, nature of the host of origin and geographical distribution (Appendix S1, Figure S7).

## 4 DISCUSSION

Is the parasitic success of *M. incognita* an evolutionary paradox? This proposition would be true only if *M. incognita* is adaptive despite having a fully parthenogenetic reproduction. Our results support these two properties.

The lack of sexual reproduction in *M. incognita* was so far only assumed based upon cytogenetic observations (Triantaphyllou, 1981, 1985) but never further supported at a molecular level. Here, the different analyses we performed at the population genomics level converge in supporting the lack of recombination and genetic exchanges in *M. incognita*. The phylogenetic network analysis based on fixed SNVs returned a bifurcating tree that separated the different isolates and not a network. This suggests a lack of genetic exchange between the isolates. In sexual “recombining” species, the mitochondrial genome accumulates mutations much faster than the nuclear genome. This is also true in the model nematode *C. elegans* where the mitochondrial mutation rate is at least two orders of magnitude higher than the nuclear mutation rate (Denver et al., 2009; Denver, Morris, Lynch, & Thomas, 2004). The higher mitochondrial accumulation of mutations is supposed to be the combined result of extremely rare or total lack of recombination, the low effective population size and the effectively haploid inheritance in mitochondria (Neiman and Taylor, 2009). In *M. incognita*, as opposed to *C. elegans*, we found that the percentage of variable positions in the mitochondrial genome is only one order of magnitude higher than in the nuclear genome. This suggests the nuclear and mitochondrial genomes evolve at a comparable rate and reinforces the idea that the nuclear genome is mostly effectively haploid and nonrecombining. Theoretically, the efficacy of selection should be lower in nonrecombining species than recombining ones. We showed that the ratio of diversity at nonsynonymous sites/ diversity at synonymous sites (*π*_n_/*π*_s_) was indeed one order of magnitude higher in *M. incognita* than in two outcrossing Caenorhabditis species. Finally, under recombination, the proportion of markers passing the 4‐gametes test should increase exponentially with physical distance while linkage disequilibrium should decrease exponentially. This was not observed in *M. incognita*, whereas a rapid exponential decrease of linkage disequilibrium was recently observed and considered an evidence for recombination in the bdelloid rotifer *Adineta vaga* (Vakhrusheva et al., 2018). Collectively, these results strongly suggest absence (or extremely rare) recombination and support the mitotic parthenogenetic reproduction of *M. incognita*.

Despite its clonal reproduction, it was already evident that *M. incognita* had an adaptive potential. Indeed, experimental evolution assays have shown the ability of *M. incognita* to overcome resistance conferred by the Mi gene in tomato in a few generations (Castagnone‐Sereno, 2006; Castagnone‐Sereno, Wajnberg, Bongiovanni, Leroy, & Dalmasso, 1994). Naturally virulent *M. incognita* populations (i.e., not controlled by the resistance gene) have also been observed in the fields and probably emerged from originally avirulent populations (Barbary, Djian‐Caporalino, Palloix, & Castagnone‐Sereno, 2015; Tzortzakakis, Conceição, Dias, Simoglou, & Abrantes, 2014; Verdejo‐Lucas, Talavera, & Andrés, 2012), although whether this resistance breaking is as rapid as under controlled laboratory conditions remains unknown. However, adapting from a compatible host plant to another very different incompatible plant is certainly more challenging than breaking down a resistance gene in a same plant. Here, we showed that the different host races defined in *M. incognita* as a function of patterns of (in)compatibilities with different plants do not follow a phylogenetic signal. This would imply multiple independent gains and losses of parasitic abilities to arrive at the current phylogenetic distribution of host compatibility patterns (i.e., host races). Whether these multiple gains and losses occurred from a hyperpolyphagous common ancestor or an ancestor with a more restricted host range remains to be clarified. To address this question, we have reconstructed host compatibilities at each ancestral node based on the SNV‐based phylogenetic classification of the *M. incognita* isolates (Figure S8). This reconstruction showed that the two hypotheses concerning the host range status of the last common ancestor were equally likely. Addition of other isolates characterized for their host race might allow favouring one or the other hypothesis in the future. Consistent with multiple gains and losses of parasitic abilities, host‐race switching within an isolate over time has already been observed. Isolates of *M. incognita* race 2 and 3, which parasitize tobacco and cotton plants, respectively, switched to behaviour similar to race 3 and 2 after staying for 8 months on coffee plants (Rui Gomes Carneiro, personal communication). Together with the previously reported ability to break down resistance gene in plants, the ability of *M. incognita* to loose and gain ability to infect different plants underlines its adaptive potential.

Overall, we provided here additional evidence for adaptability and the first whole‐genome level assessment for the lack of recombination in *M. incognita*, consolidating this species as a main model to study the paradox of adaptability and parasitic success in the absence of sexual reproduction.

The adaptability of *M. incognita* despite its obligatory asexual reproduction and the lack of phylogenetic signal underlying the host races have important practical implications at the agricultural level. Characterizing populations that differ in their ability to infest a particular host (that carries specific resistance genes) is of crucial importance for growers and agronomists. Indeed, the main *Meloidogyne* spp. control strategies consist in deploying resistant cultivars and appropriate crop rotation against a specific given race. If the identity of a population is unknown, the crop selected for use in a management scheme may cause dramatic increases in nematode populations (Hartman & Sasser, 1985). However, the adaptability of *M. incognita* casts serious doubts on the durability of such strategies and must be taken into account in rotation schemes. Furthermore, the biological reality of host races themselves is challenged by the lack of underlying genetic signal. Actually, the initial host‐race concept has never been universally accepted, in part because it covered only a small portion of the whole potential variation in parasitic ability (Moens et al., 2009). Although *M. incognita* was already known to parasitize hundreds of host plants, only six different host standards were used to characterize four races. New host races might be defined in the future when including additional hosts in the differential test. Furthermore, using the same six initial host plant species, two additional *M. incognita* races that did not fit into the previously published race scheme have already been described (Robertson et al., 2009). Although the terminology “races” of *Meloidogyne* spp. has been recommended not to be used since 2009 (Moens et al., 2009), several papers related to *M. incognita* diversity of host compatibility or selection of resistant cultivars are still using this term, including on coffee (Lima et al., 2015; Peres et al., 2017); cotton (Mota et al., 2013; da Silva et al., 2014); or soybean (Fourie, McDonald, & Waele, 2006). This reflects the practical importance to differentiate *M. incognita* populations according to their different ranges of host compatibilities. However, because these variations in host ranges are not monophyletic and thus do not follow shared common genetic ancestry, we recommend abandoning the term “race”. Terms like “pathotype” or “biotype” that only refer to a phenotype and do not imply an underlying phylogenetic signal should be preferred (Sturhan, 1985).

What level of intraspecific genome polymorphism is required to cover the different ranges of host compatibilities in *M. incognita* and their ability to survive in different environments, despite their clonal reproduction? In this study, we found that the cumulative fixed divergence across the eleven isolates from Brazil and the reference genome (sampled initially from Mexico) reached ~ 0.02% of the nucleotides. Addition of isolates from Africa, the French Antilles and the United States did not increase the maximal divergence. This relatively low divergence is rather surprising, considering the variability in terms of distinct compatible host spectra (host races). Host‐specific SNV were found only for Race 2 and no functional consequence for these SNV could be found, as they did not fall in predicted coding or evident regulatory regions. Furthermore, the existence of race‐specific SNV themselves is even questionable as addition of other isolates might disqualify the few Race 2‐specific SNPs in the future. Similarly, there were no disruptive variations identified in the coding regions matching the current host plants, we found no SNV specifically associated to cotton, only one synonymous variant for soybean and only one synonymous variant for tobacco.

Collectively, our observations indicate that *M. incognita* is versatile and adaptive despite its clonal mode of reproduction. The relatively low divergence at the SNV level suggests acquisition of point and short‐scale mutations followed by selection of the fittest haplotype is probably not the main or at least not the sole player in the adaptation of this species to different host plants and environments. Other mechanisms such as epigenetics, copy number variations, movement of transposable elements or large‐scale structural variations could be at play in the surprising adaptability of this clonal species. Consistent with this idea, convergent gene copy number variations (CNV) have recently been associated with breaking down of a resistance by *M. incognita* (Castagnone‐Sereno et al., 2019). Interestingly, the parthenogenetic marbled crayfish has multiplied by more than 100 its original area of repartition across Madagascar, adapting to different environments despite showing a surprisingly low number of nucleotide variation (only ~400 SNV on a ~3 Gb genome representing a proportion of variable positions of 1.3 × 10^−7^ only). This also led the authors to suggest that mechanisms other than acquisition of point mutations and selection of the fittest haplotype must be involved (Gutekunst et al., 2018).

Previously, we have shown that the genome structure of *M. incognita* itself could participate in its versatility. Indeed, being allopolyploid, *M. incognita* has >90% of its genes in multiple copies. The majority of these gene copies showed diverged expression patterns one from the other and signs of positive selection between the gene copies have been identified (Blanc‐Mathieu et al., 2017). How the expression patterns of these gene copies vary across different geographical isolates with different host compatibilities would be interesting to explore in the future.

## CONFLICT OF INTEREST

The authors declare that they have no financial conflict of interest with the content of this article.

## AUTHOR CONTRIBUTIONS

EGJD, PC‐S, GDK, EVSA and LD contributed to the design of the project; RMDGC, ACZM, EM contributed to collection of samples. Classification of samples into races was done by RMDGC, ACZM, EM; library preparations were performed by M‐JA; GDK, EGJD, LD, EVSA, MBB and DKK contributed to the analysis and interpretation of data. EGJD and GDK wrote the manuscript with contributions of EVSA, RMDGC, PC‐S, M‐JA and LD. All authors approved the final manuscript.

## Data Availability

All the sequence data generated during this study have been deposited at the NCBI under GEO accession GSE116847 and available at this URL: https://www.ncbi.nlm.nih.gov/geo/query/acc.cgi?acc=GSE116847. The different scripts and R codes used to process the data are available on GitHub at the following URL: https://github.com/GDKO/gdk_scripts/tree/master/popgenvcf
