# Supplementary material for: Population genomics supports clonal reproduction and multiple independent gains and losses of parasitic abilities in the most devastating nematode pest
Source: Evol Appl. 2019 Nov 6;13(2):442–57. doi: 10.1111/eva.12881 (PMC6976969; doi:10.1111/eva.12881)
Supplement: Supplementary file 1 [file EVA-13-442-s001.pdf]

SUPPLEMENTARY MATERIAL

Population genomics supports clonal reproduction and multiple independent gains and losses of parasitic abilities in the most devastating nematode pest

Georgios D. Koutsovoulos[1*], Eder Marques[2], Marie-Jeanne Arguel[3], Laurent Duret[4], Andressa C.Z. Machado[5], Regina M.D.G. Carneiro[2], Djampa K. Kozlowski[1], Marc Bailly-Bechet[1], Philippe Castagnone-Sereno[1], Erika V.S. Albuquerque[2*], Etienne G.J. Danchin[1*]

[1]INRA, CNRS, Université Côte d'Azur, ISA, France

[2]Embrapa Recursos Genéticos e Biotecnologia, Brasília, Brasil

[3]CNRS, Université Côte d'Azur, IPMC, France

[4]Université de Lyon, Université Lyon 1, CNRS, Laboratoire de Biométrie et Biologie Evolutive UMR 5558, F-69100, Villeurbanne, France

[5]Área de Proteção de Plantas, Instituto Agronômico do Paraná, Londrina, Paraná, Brasil

Corresponding author: Etienne GJ Danchin, etienne.danchin@inra.fr

* equal contributions

Species	Tobacco<br>*NC 95*	Tomato<br>*Rutgers*	Watermelon<br>*Charleston Gray*	Peanut<br>*Florunner*	Pepper<br>*Early California Wonder*	Cotton<br>*Deltapine 61*
M. incognita						
Race 1	-	+	+	-	+	-
Race 2	+	+	+	-	+	-
Race 3	-	+	+	-	+	+
Race 4	+	+	+	-	+	+
M. arenaria						
Race 1	+	+	+	+	+	-
Race 2	+	+	+	-	-	-
M. javanica	+	+	+	-	-	-
M. hapla	+	+	-	+	+	-

Table S1. Expected host reactions of the NCDHT to discriminate *M. incognita* races.

Nematode reproduction indexes (average of 4 replicates) were used to determine cotton and tobacco as good/susceptible (+) or poor/resistant (-) hosts. Tobacco and cotton are highlighted (light grey shadow) to show that the differentiation of the four *M. incognita* races is determined by the combined reaction of these two hosts alone.

	Raw		After Trimming			
Isolate	PE reads #	No. of bases	PE reads #	No. of bases	Coverage	Mapped (%)
R1-2	77.0E+06	23.1E+09	76.4E+06	22.7E+09	122.53	97.39
R1-3	76.2E+06	22.9E+09	75.5E+06	22.4E+09	121.20	99.51
R1-6	75.8E+06	22.8E+09	75.0E+06	22.3E+09	120.55	97.28
R2-1	75.7E+06	22.7E+09	75.1E+06	22.3E+09	120.41	98.96
R2-6	76.0E+06	22.8E+09	75.3E+06	22.4E+09	121.01	94.26
R3-1	75.1E+06	22.5E+09	74.5E+06	22.1E+09	119.51	89.34
R3-2	75.3E+06	22.6E+09	74.7E+06	22.2E+09	119.92	98.16
R3-4	75.3E+06	22.6E+09	74.6E+06	22.2E+09	119.79	98.47
R4-1	75.6E+06	22.7E+09	75.1E+06	22.3E+09	120.52	99.04
R4-3	75.8E+06	22.8E+09	75.2E+06	22.4E+09	120.89	97.00
R4-4	75.6E+06	22.7E+09	75.0E+06	22.3E+09	120.46	99.32

Table S2. Statistics of generated sequence data and genome coverage per isolate.

Number of Illumina paired-end reads and amount of bases generated per isolate. Values indicated before and after quality filtering (trimming). Coverage values of mapped reads are given according to a reference genome size of 184 Mb.

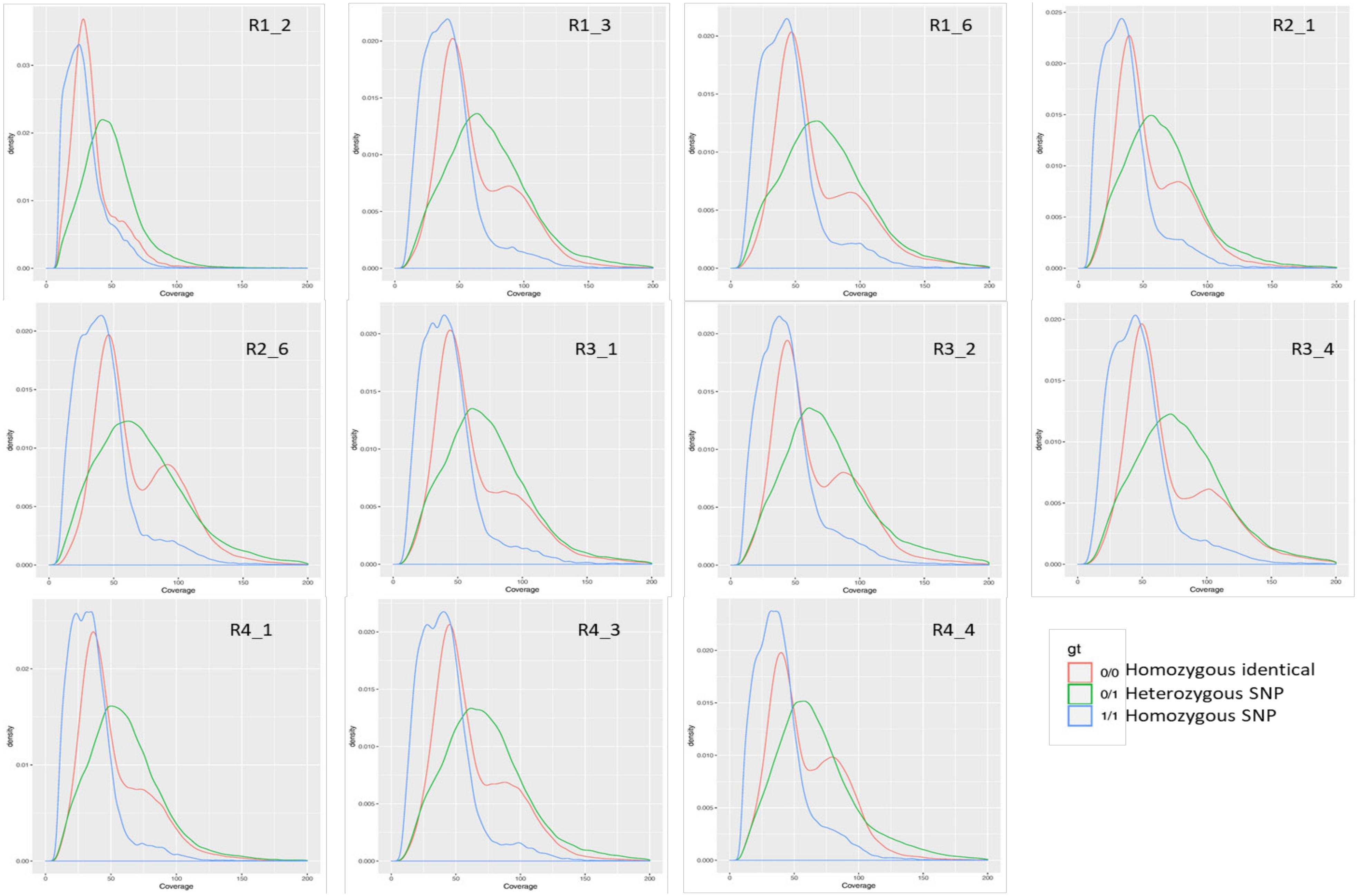

Fig. S1. Distribution of base coverage per category of SNP on the reference genome for each isolate.

Density plot of base coverage for homozygous identical (0/0) heterozygous SNP (0/1) and homozygous SNP.

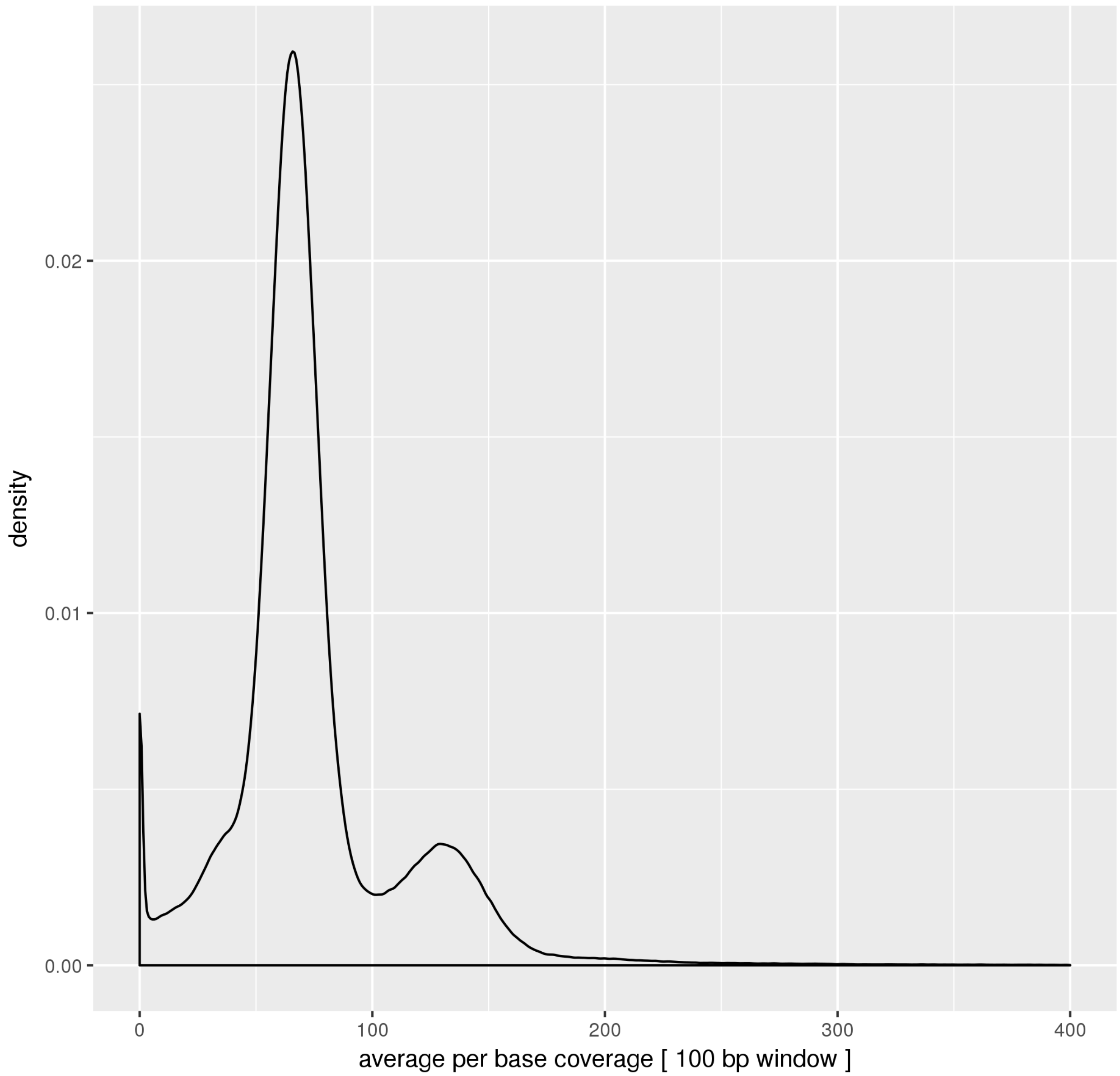

Fig. S2. Distribution of average per base coverage in 100bp windows on the *M. incognita* reference genome.

Illumina reads used to produce the reference *M. incognita* genome assembly (Blanc-Mathieu et al., 2017) were mapped back to the reference genome using the same methodology as for the 11 isolates. Average per base coverage for each 100bp window was counted and the density at each coverage was plotted.

Effector Name	GENE	POS	REF	ALT	TYPE	R1-2	R1-3	R1-6	R2-1	R2-6	R3-1	R3-2	R3-4	R4-1	R4-3	R4-4	Annotation
**Dorsal Gland**																	
4D03	Minc3s00043g02427	172356	T	A	snp	1	0	0	0	0	0	0	0	0	0	0	A|missense_variant|Minc3s00043g02427|1/4|c.43T>A|p.Leu15Ile
4D03	Minc3s00043g02427	172361	C	A	snp	1	0	0	0	0	0	0	0	0	0	0	A|missense_variant|Minc3s00043g02427|1/4|c.48C>A|p.Phe16Leu
4D03	Minc3s00043g02427	172378	TAAATG	CTAATA	complex	1	0	0	0	0	0	0	0	0	0	0	CTAATA|missense_variant|Minc3s00043g02427|1/4|c.65_70delTAAATGinsCTAATA|p.LeuAsnVal22SerAsnIle
4D03	Minc3s00043g02427	172426	A	T	snp	1	0	0	0	0	0	0	0	0	0	0	T|missense_variant|Minc3s00043g02427|1/4|c.113A>T|p.Tyr38Phe
4D03	Minc3s00043g02427	172482	TCTTTACCA	ATTTTGCCG	complex	1	0	0	0	0	0	0	0	0	0	0	ATTTTGCCG|missense_variant|Minc3s00043g02427|1/4|c.169_177delTCTTTACCAinsATTTTGCCG|p.Ser57Ile
4D03	Minc3s00043g02427	172530	ATTTTTTGCTTACAGT	ATTTTTTTGATTAAAGC	complex	1	0	0	0	0	0	0	0	0	0	0	TGATTAAAGC|frameshift_variant&splice_acceptor_variant&missense_variant&splice_region_variant&intron_variant|Minc3s00043g02427|2/4|c.186-8_186delGCTTACAGTinsTGATTAAAGC|p.Phe62fs
4D03	Minc3s00043g02427	172578	CG	TA	mnp	1	0	0	0	0	0	0	0	0	0	0	TA|missense_variant|Minc3s00043g02427|2/4|c.219_220delCGinsTA|p.Val74Ile
9H10	Minc3s00815g17694	6866	A	G	snp	0	0	0	0	0	0	1	0	0	0	0	G|missense_variant|Minc3s00815g17694|2/4|c.143A>G|p.Lys48Arg
9H10	Minc3s00815g17694	6916	CGTGATGAAGTTG	GGTATTGAGGAAT	complex	0	.	0	0	0	0	1	0	0	.	0	GGTATTGAGGAAT|missense_variant|Minc3s00815g17694|2/4|c.193_205delCGTGATGAAGTTGinsGGTATTGAGGAAT|p.ArgAspGluValAsp65GlyIleGluGluTyr
Minc12754	Minc3s00905g18741	30878	A	C	snp	0	0	0	0	0	0	1	0	0	0	1	C|missense_variant|Minc3s00905g18741|2/2|c.110T>G|p.Leu37Arg
25B10	Minc3s03649g34419	6707	C	T	snp	1	1	1	0	0	0	0	0	0	1	0	T|missense_variant|Minc3s03649g34419|1/3|c.263G>A|p.Gly88Glu
25B10	Minc3s03649g34419	6713	A	G	snp	1	0	1	1	0	0	0	0	0	1	0	G|missense_variant|Minc3s03649g34419|1/3|c.257T>C|p.Ile86Thr
25B10	Minc3s03649g34419	6758	GTCAA	ATCAG	complex	1	0	1	1	0	0	1	1	0	1	1	ATCAG|missense_variant|Minc3s03649g34419|1/3|c.208_212delTTGACinsCTGAT|p.Thr71Ile
25B10	Minc3s03649g34419	6897	C	T	snp	1	0	1	1	0	1	1	0	0	1	0	T|missense_variant|Minc3s03649g34419|1/3|c.73G>A|p.Gly25Ser
25B10	Minc3s03649g34419	6959	A	G	snp	1	0	1	1	0	1	0	0	0	1	0	G|missense_variant|Minc3s03649g34419|1/3|c.11T>C|p.Val4Ala

Effector Name	GENE	POS	REF	ALT	TYPE	R1-2	R1-3	R1-6	R2-1	R2-6	R3-1	R3-2	R3-4	R4-1	R4-3	R4-4	Annotation
**Subventral Gland**																	
Minc03325	Minc3s00020g01295	149823	A	G	snp	1	0	0	0	0	0	0	0	0	0	0	G|missense_variant|Minc3s00020g01295|6/6|c.1309A>G|p.Asn437Asp
Minc03328	Minc3s00020g01299	174234	C	T	snp	0	1	0	0	0	0	0	0	0	0	0	T|missense_variant|Minc3s00020g01299|5/6|c.817G>A|p.Asp273Asn
Minc01696	Minc3s00036g02098	45132	CC	CG	snp	1	0	0	0	0	0	0	0	0	0	0	CG|missense_variant|Minc3s00036g02098|3/8|c.355C>G|p.Gln119Glu
Minc01696	Minc3s00036g02098	47885	T	G	snp	0	0	0	0	0	0	1	0	0	0	0	G|missense_variant|Minc3s00036g02098|8/8|c.1677T>G|p.Ile559Met
Mi-Xyl1	Minc3s00084g04025	91786	CA	TT	complex	1	0	0	0	0	0	0	0	0	0	0	TT|missense_variant|Minc3s00084g04025|1/2|c.60_61delTGinsAA|p.Asp21Asn
8E08B	Minc3s00139g05823	106553	A	C	snp	0	0	0	1	0	0	0	0	0	0	0	C|missense_variant|Minc3s00139g05823|3/5|c.708T>G|p.Ile236Met
8E08B	Minc3s00139g05823	106852	G	C	snp	0	0	0	1	0	0	0	0	0	0	0	C|missense_variant|Minc3s00139g05823|3/5|c.409C>G|p.Gln137Glu
30G11	Minc3s00271g09100	84986	T	C	snp	0	0	0	0	0	0	0	0	1	0	0	C|missense_variant|Minc3s00271g09100|1/10|c.22T>C|p.Phe8Leu
30G11	Minc3s00271g09100	84997	A	C	snp	0	0	0	0	0	0	0	0	1	0	0	C|missense_variant|Minc3s00271g09100|1/10|c.33A>C|p.Leu11Phe
Minc08073, Minc10418	Minc3s00371g11166	42606	ACCGATG	GCCAATA	complex	0	0	0	0	0	0	1	0	0	0	1	GCCAATA|missense_variant|Minc3s00371g11166|1/12|c.6_12delACCGATGinsGCCAATA|p.Met4Ile
Minc08073, Minc10418	Minc3s00371g11166	42755	C	T	snp	0	0	0	0	0	0	0	0	0	0	1	T|missense_variant|Minc3s00371g11166|1/12|c.155C>T|p.Ala52Val
Minc08073, Minc10418	Minc3s00371g11166	42868	T	A	snp	0	0	0	0	0	0	1	0	0	0	1	A|missense_variant|Minc3s00371g11166|1/12|c.268T>A|p.Phe90Ile
Minc08073, Minc10418	Minc3s00371g11166	43365	ATATC	CTATT	complex	0	0	0	0	0	0	1	0	0	0	1	CTATT|missense_variant|Minc3s00371g11166|4/12|c.629_633delATATCinsCTATT|p.His210Pro
Minc08073, Minc10418	Minc3s00371g11166	43733	A	G	snp	0	0	0	0	0	0	0	0	0	0	1	G|missense_variant|Minc3s00371g11166|6/12|c.901A>G|p.Arg301Gly
Minc08073, Minc10418	Minc3s00371g11166	43967	CG	TA	mnp	0	0	0	0	0	0	0	0	0	0	1	TA|missense_variant|Minc3s00371g11166|7/12|c.1091_1092delCGinsTA|p.Ala364Val
5G06	Minc3s00800g17511	23637	C	T	snp	1	1	1	1	1	1	1	1	1	1	1	T|missense_variant|Minc3s00800g17511|2/8|c.125G>A|p.Arg42Gln
5G06	Minc3s00800g17511	23758	T	G	snp	1	1	1	1	1	1	1	1	1	1	1	G|missense_variant|Minc3s00800g17511|1/8|c.45A>C|p.Leu15Phe
5G06	Minc3s00800g17511	23795	A	T	snp	1	1	1	1	1	1	1	1	1	1	1	T|missense_variant|Minc3s00800g17511|1/8|c.8T>A|p.Phe3Tyr
Mi-PG1	Minc3s00861g18203	45073	A	G	snp	1	0	0	0	0	0	0	0	0	0	0	G|missense_variant|Minc3s00861g18203|3/8|c.373T>C|p.Phe125Leu
Mi-PG1	Minc3s00861g18203	45138	G	A	snp	1	0	0	0	0	0	0	0	0	0	0	A|missense_variant|Minc3s00861g18203|3/8|c.308C>T|p.Thr103Ile
Mi-PG1	Minc3s00861g18203	45147	G	A	snp	1	0	0	0	0	0	0	0	0	0	0	A|missense_variant|Minc3s00861g18203|3/8|c.299C>T|p.Ala100Val
Mi-PG1	Minc3s00861g18203	45581	C	T	snp	1	0	0	0	0	0	0	0	0	0	0	T|missense_variant|Minc3s00861g18203|2/8|c.119G>A|p.Ser40Asn
Mi-VAP2	Minc3s01051g20218	36189	CTG	TTA	complex	0	0	1	0	1	1	0	0	0	0	1	TTA|missense_variant|Minc3s01051g20218|4/4|c.756_758delCAGinsTAA|p.Ser253Asn

Minc10418, Minc08073	Minc3s01112g20831	17736	C	T	snp	0	0	0	0	0	0	1	0	0	0	1	T|missense_variant|Minc3s01112g20831|1/12|c.155C>T|p.Ala52Val
Minc10418, Minc08073	Minc3s01112g20831	17953	T	A	snp	0	0	0	0	0	0	1	0	0	0	1	A|missense_variant|Minc3s01112g20831|1/12|c.372T>A|p.Phe124Leu
Minc10418, Minc08073	Minc3s01112g20831	18956	CG	TA	mnp	0	0	0	0	0	0	1	0	0	0	1	TA|missense_variant|Minc3s01112g20831|7/12|c.1091_1092delCGinsTA|p.Ala364Val
Minc10418, Minc08073	Minc3s01112g20831	19451	A	G	snp	0	0	0	0	0	0	1	0	0	0	1	G|missense_variant|Minc3s01112g20831|9/12|c.1486A>G|p.Thr496Ala
Minc10418, Minc08073	Minc3s01112g20831	19459	ATCTAAAT	GTCGAAAG	complex	0	0	0	0	0	0	1	0	0	0	1	GTCGAAAG|missense_variant|Minc3s01112g20831|9/12|c.1494_1501delATCTAAATinsGTCGAAAG|p.Leu501Val
Minc10418, Minc08073	Minc3s01112g20831	19670	A	T	snp	0	0	0	0	0	0	1	0	0	0	1	T|missense_variant|Minc3s01112g20831|10/12|c.1629A>T|p.Glu543Asp
Minc15401	Minc3s06052g39242	4585	A	C	snp	1	.	1	1	0	.	.	1	1	.	1	C|missense_variant|Minc3s06052g39242|1/1|c.795A>C|p.Gln265His

Table S3. Non-synonymous SNV in effector genes

Missense variants falling in coding regions of effector genes known to be specifically expressed in the nematode secretory dorsal or sub-ventral gland cells.

Appendix S1. Test for association between SNP-based clusters and biological traits

We tested for significant association between the SNP-based clusters of the different *M. incognita* isolates and the following biological traits:

- Pattern of host compatibility (= host races)
- Nature of the crop plant of origin at the moment of isolation
- Geographical distribution

For the two first above-mentioned biological traits we used a Fisher's exact test while for geographical distance, we used Isolation By Distance (IBD) analysis. All these analyses were performed based on the data gathered in the Table S4 (below).

Name	Cluster	Race	Host	Country	Region	City	GPS coordinates
R1-2	A	1	Soybean	Brazil	PR	Londrina	23°18'22.0"S 51°10'07.9"W
R3-2	B	3	Soybean	Brazil	PR	Londrina	23°18'22.0"S 51°10'07.9"W
R4-4	B	4	Cotton	Brazil	SP	Vargem Grande do Sul	21°49'52.9"S 46°53'24.3"W
R2-1	C	2	Tobacco	Brazil	SC	Sombrio	29°05'54.0"S 49°37'50.5"W
R2-6	C	2	Coffee	Brazil	PR	São Jorge do Patrocínio	23°43'47.2"S 53°56'38.4"W
R1-3	C	1	Cucumber	Brazil	SP	Piracicaba	22°44'05.1"S 47°38'48.2"W
R1-6	C	1	Tobacco	Brazil	PR	Mercedes	24°27'13.1"S 54°10'04.5"W
R4-3	C	4	Watermelon	Brazil	PR	Londrina	23°18'22.0"S 51°10'07.9"W
R3-1	C	3	Cotton	Brazil	PR	Umuarama	23°46'22.0"S 53°18'34.4"W
R4-1	C	4	Cotton	Brazil	MT	Campo Verde	15°33'32.0"S 55°10'04.3"W
R3-4	C	3	Cotton	Brazil	PR, MS, BA	Umuarama, Londrina, Dourados, L.E. Magalhães	NA: pool of species
A14	A	NA	Tomato	Libya	North East	NA	32°43'51.6"N 22°07'47.4"E
L9	A	NA	NA	Ivory Coast	NA	NA	7°42'42.4"N 5°02'17.4"W
L19	A	NA	NA	French West Indies	Guadeloupe	NA	16°12'26.0"N 61°40'13.1"W
Morelos	C	3	NA	Mexico	Morelos	Morelos	18°45'11.1"N 99°06'29.7"W
L27	C	1	NA	USA	NA	NA	NA: US state not known
W1	C	NA	Tomato	USA	California	Yolo County	38°44'22.1"N 121°48'35.4"W
557R	C	NA	Tomato	USA	North Carolina	NA	35°49'30.3"N 78°39'11.9"W
HarC	C	NA	Grape	USA	California	NA	34°13'03.2"N 117°32'54.8"W
VW6	C	NA	Cotton	USA	California	NA	34°13'03.2"N 117°32'54.8"W

Table S4. Cluster assignment, host race status, host of origin and geographical origin of the 11 isolates.

Association between genetic clusters and host races

The Fisher's exact test checks for independency between the tested variables, based on the co-occurrence counts.

We used the following contingency table of the 13 isolates (including the L27 isolate characterized as Race 1 and the Morelos isolate characterized as a Race 3) in clusters and host races:

Cluster / Host Race	R1	R2	R3	R4
A	1	0	0	0
B	0	0	1	1
C	3	2	3	2

The Fisher exact test returns a p-value of 1 indicating no significant association between host races and SNP clusters of isolates.

To evaluate the sensitivity of the Fisher's exact test, we also ran the same test on an artificially modified contingency table. In this modified contingency table, we assigned all the R3 to cluster B and all the R4 to cluster C and left the rest of the table unchanged.

Cluster / Host Race	R1	R2	R3	R4
A	1	0	0	0
B	0	0	4	0
C	3	2	0	3

This modification alone was enough to obtain a p-value of 0.007, indicating that this method is sensitive in detecting significant association between genetic clusters and biological traits.

Association between genetic clusters and the nature of the crop of origin

For crop species, we performed two analyses.

- 1. All the isolates for which a crop species of origin was documented regardless the number of occurrences of the crop species (16 isolates on 8 different crops cf. Table S4).

Cluster	Coffee	Cotton	Cucumber	Grape	Soybean	Tobacco	Tomato	Watermel
A	0	0	0	0	1	0	1	0
B	0	1	0	0	1	0	0	0
C	1	4	1	1	0	2	2	1

The Fisher exact test returns a p-value of 0.69, indicating no significant association between crop species and clusters

- 2. All the isolate / crop species associations where host species count was >1 to eliminate potential biases associated to single isolate crop species (12 isolates on 4 different crops c.f. Table S4)

Cluster	Cotton	Soybean	Tobacco	Tomato
A	0	1	0	1
B	1	1	0	0
C	4	0	2	2

The Fisher exact test returns a p-value of 0.26, indicating, here again, no significant association between crop species and clusters.

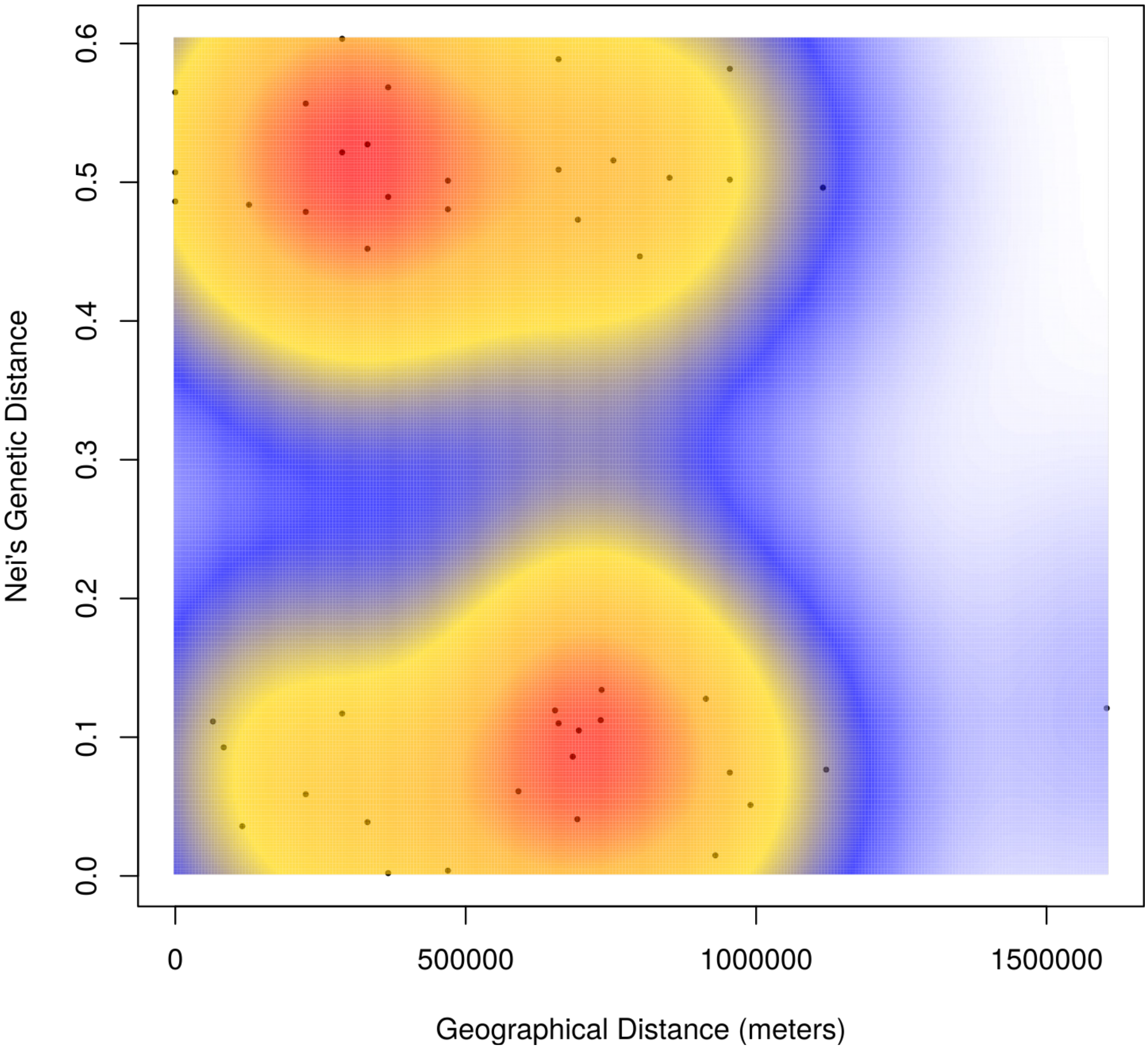

Fig. S3. Isolation by Distance (IBD) analysis between the 11 Brazilian isolates.

Each pair of isolates is plotted in a scatterplot were the x-axis is the geographical distance and the y-axis the genetic distance. Density of points is represented by different colours (blue - low, red - high).

	Cluster A	Cluster B	Cluster C
Cluster A	X	0.98088	0.3523
Cluster B	0.9904	X	0.52805
Cluster C	0.83675	0.84255	X

Table S5. Fixation index (F_{ST}) based on homozygous SNPs (upper part mean values, lower part weighted values).

Fig. S5

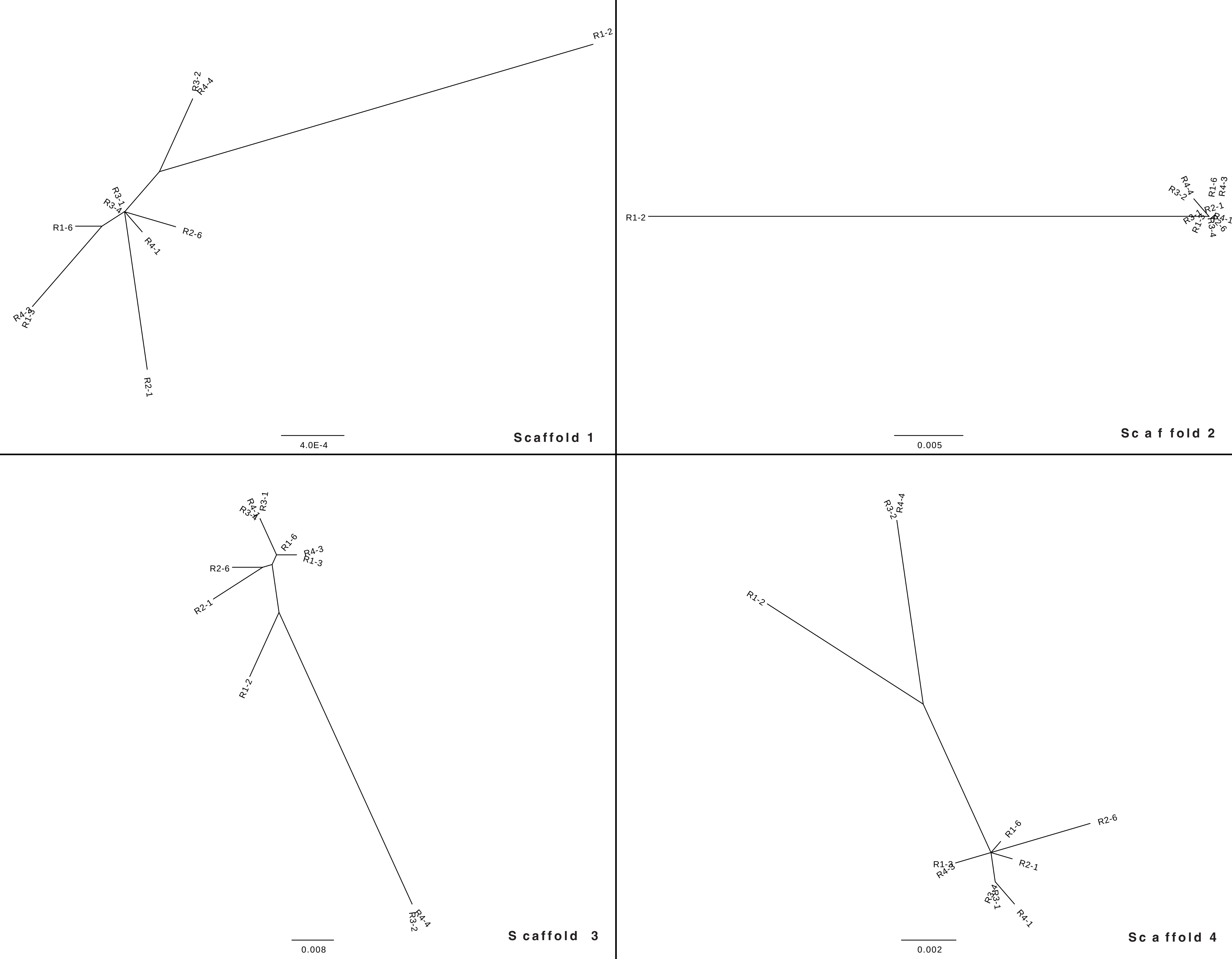
R1-2
R3-2
R4-4
R3-1
R3-4
R1-6
R2-6
R4-1
R4-3
R1-3
R2-1
4.0E-4
Scaffold 1
0.005
Scaffold 2
0.008
Scaffold 3
0.002
Scaffold 4

Fig. S5

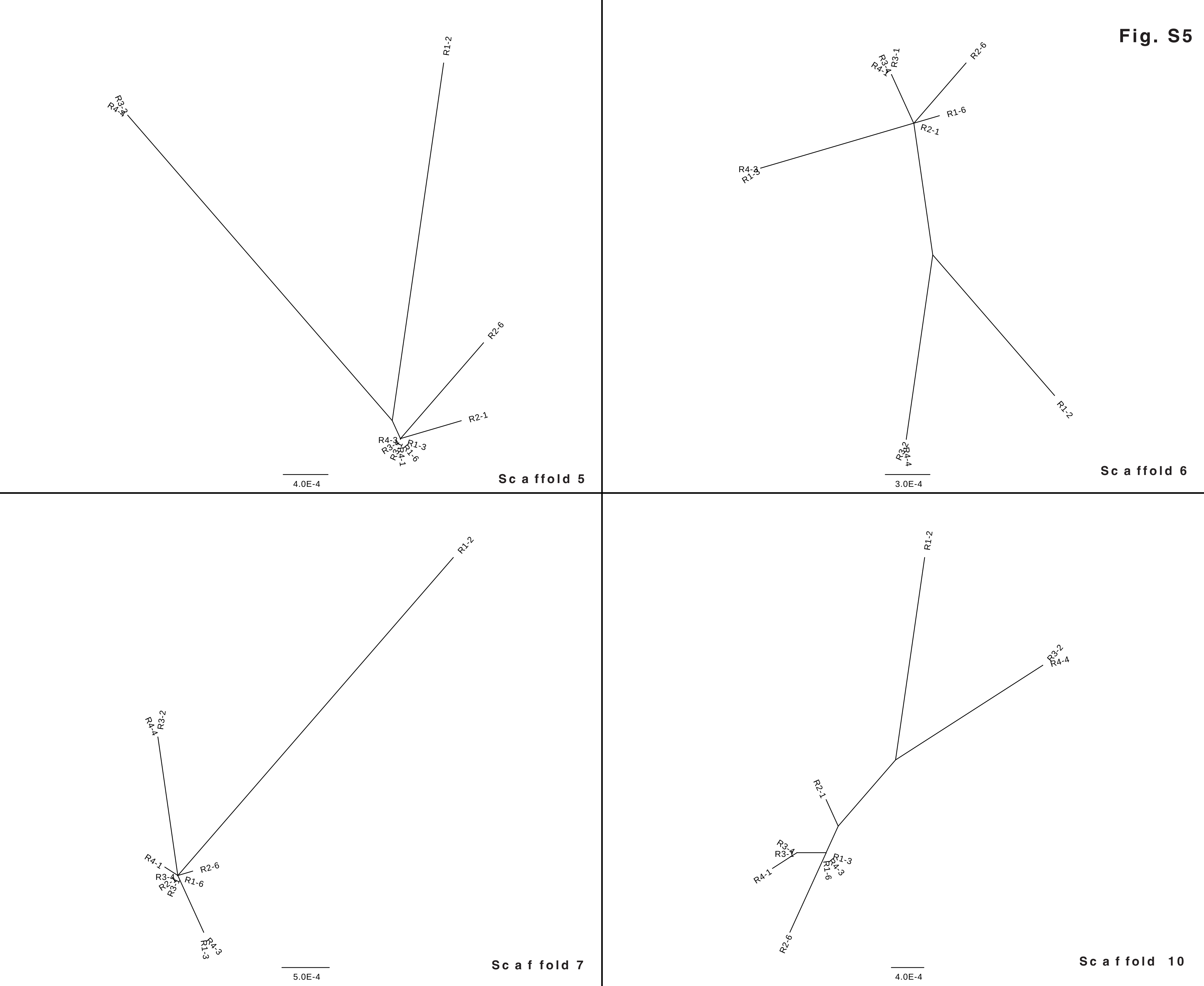
R1-2
R3-2
R4-4
R2-6
R2-1
R4-3
R1-3
R1-6
R3-4
R4-1
4.0E-4
Scaffold 5
R3-1
R1-6
R2-1
R4-3
R1-3
R3-2
R4-4
R1-2
3.0E-4
Scaffold 6
R1-2
R3-2
R4-4
R4-1
R2-6
R3-4
R1-6
R4-3
R1-3
5.0E-4
Scaffold 7
R1-2
R3-2
R4-4
R2-1
R3-4
R3-1
R1-3
R4-3
R1-6
R4-1
R2-6
4.0E-4
Scaffold 10

Fig. S5

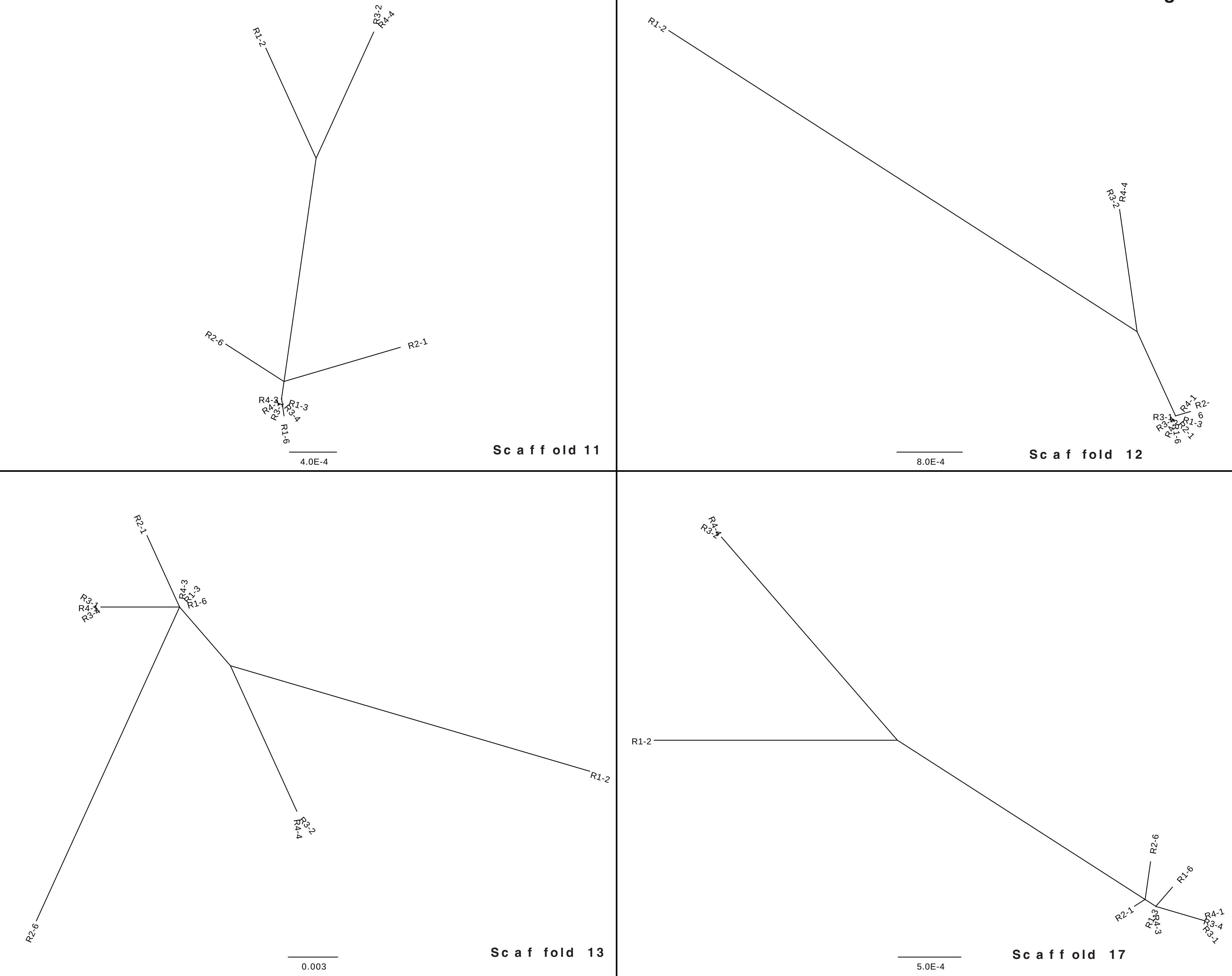
R1-2
R3-2
R4-4
R2-6
R2-1
R1-6
4.0E-4
Scaffold 11
R1-2
R3-2
R4-4
R4-1
8.0E-4
Scaffold 12
R2-1
R4-3
R1-3
R1-6
R1-2
R3-2
R4-4
R2-6
0.003
Scaffold 13
R1-2
R2-6
R1-6
R2-1
R4-1
R3-4
R3-1
5.0E-4
Scaffold 17

Fig. S5

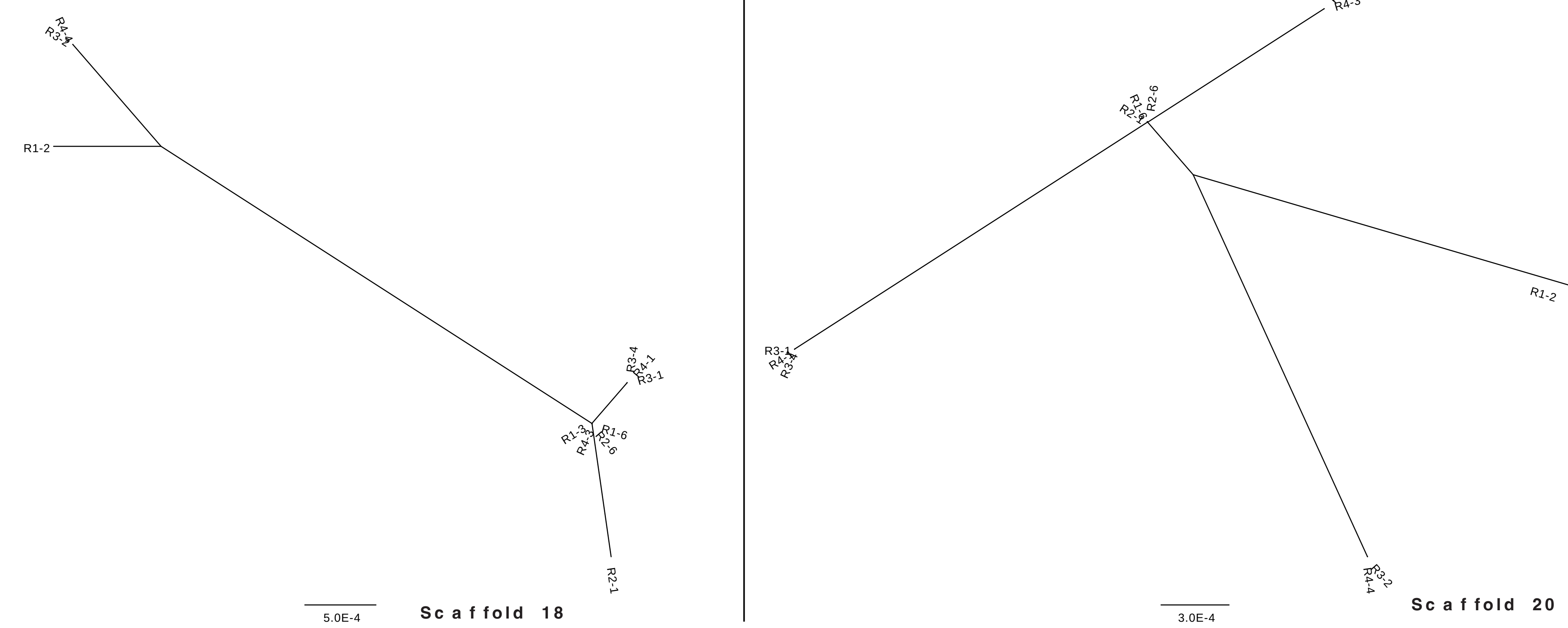
R4-4
R3-2
R1-2
R3-4
R4-1
R3-1
R1-3
R4-3
R1-6
R2-6
R2-1
5.0E-4
Scaffold 18
R1-3
R4-3
R2-6
R1-6
R2-1
R1-2
R3-1
R4-1
R3-4
R3-2
R4-4
3.0E-4
Scaffold 20

Fig. S5. Phylogenetic analysis based on SNPS present in the 14 biggest scaffolds with enough variable positions in coding regions

Unrooted topologies of all the ML trees constructed from SNPs present in coding regions for the 14 longest scaffolds containing such SNPs in enough abundance.

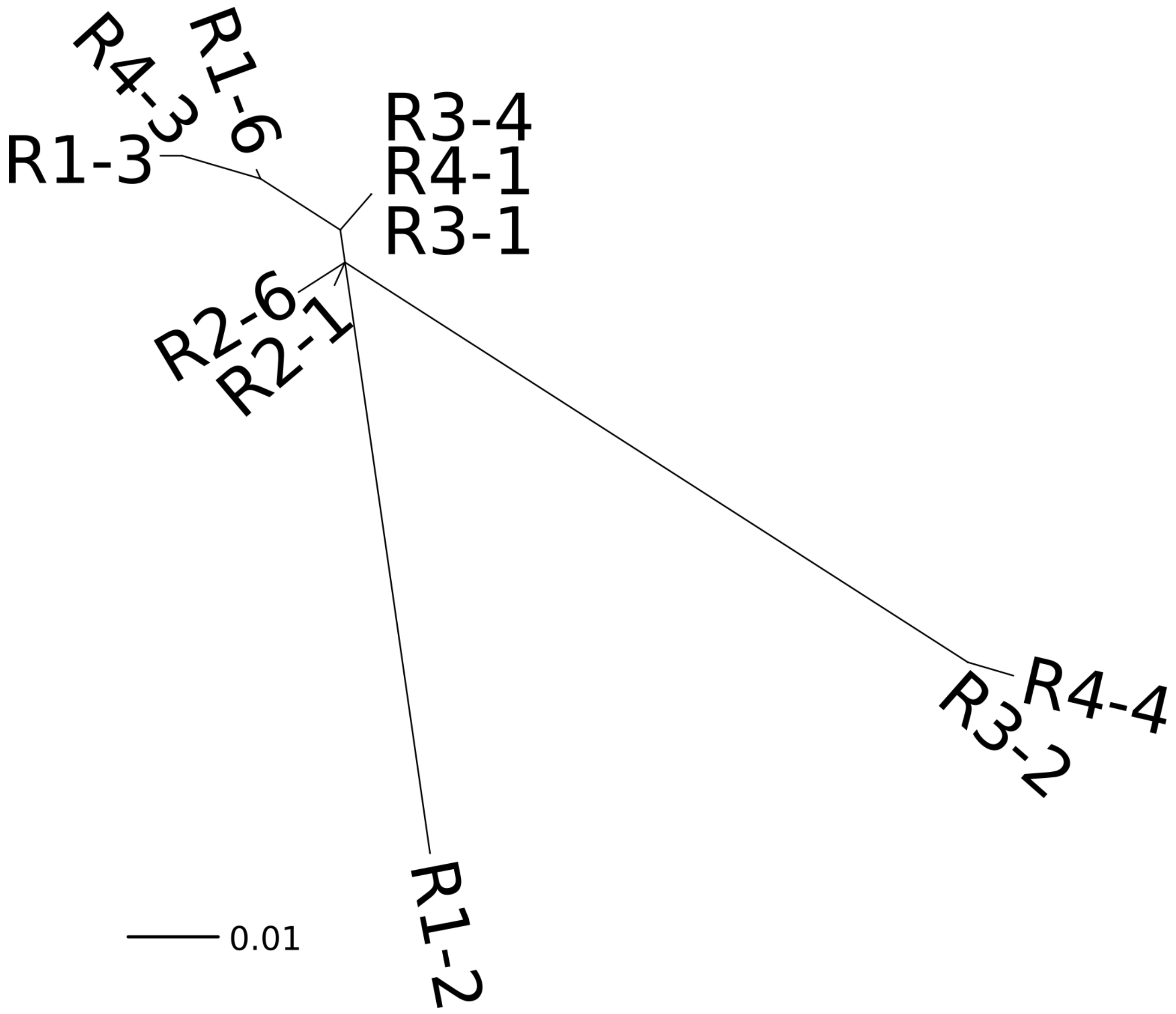

Fig. S6. Phylogenetic analysis based on SNPs in the mitochondrial genome

The tree topology exactly recapituclates the one obtained at the nuclear level.

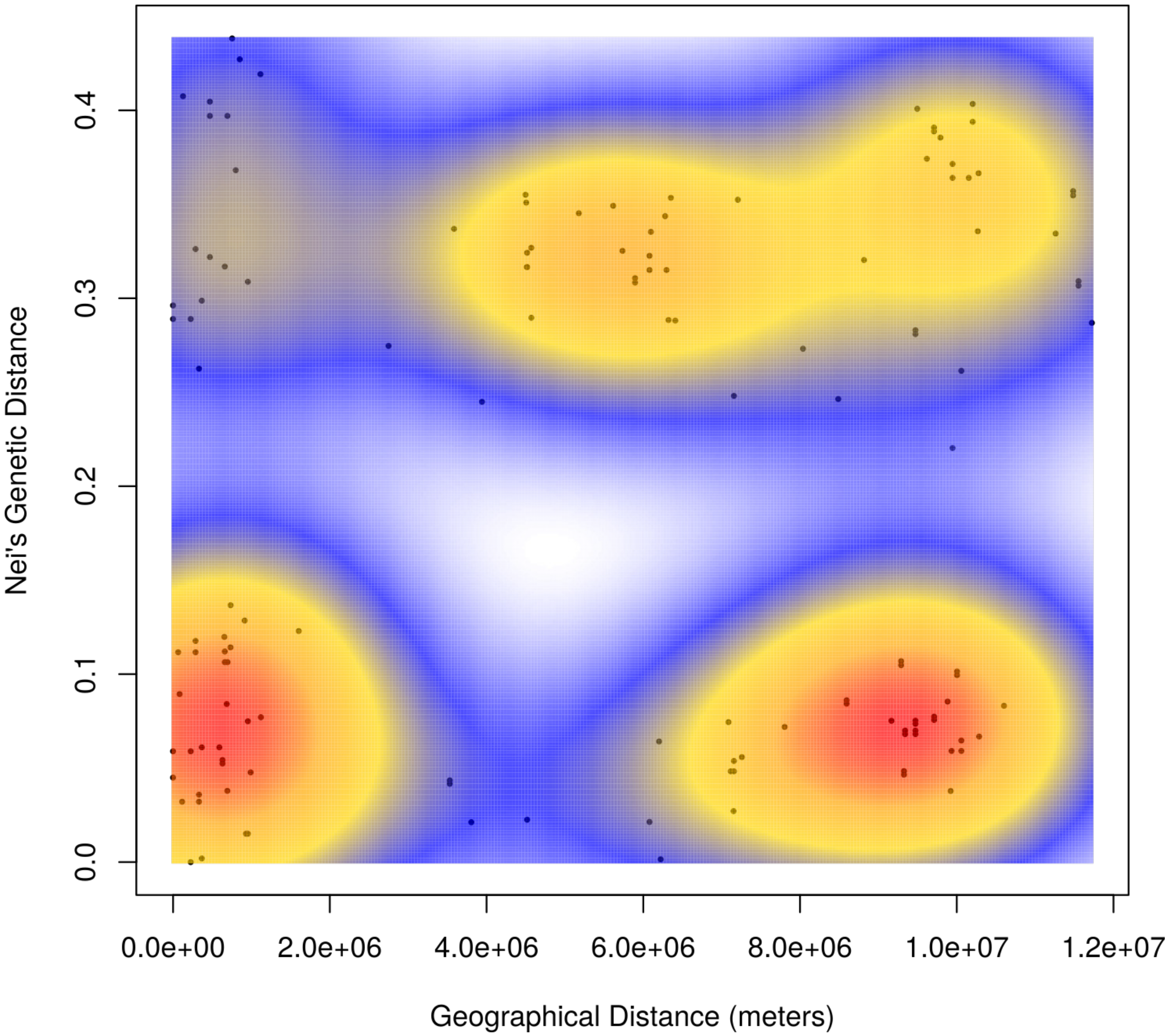

Fig. S7. Isolation by Distance (IBD) analysis between all the *M. incognita* isolates available worldwide.

Each pair of isolates is plotted in a scatterplot were the x-axis is the geographical distance and the y-axis the genetic distance. Density of points is represented by different colours (blue - low, red - high).

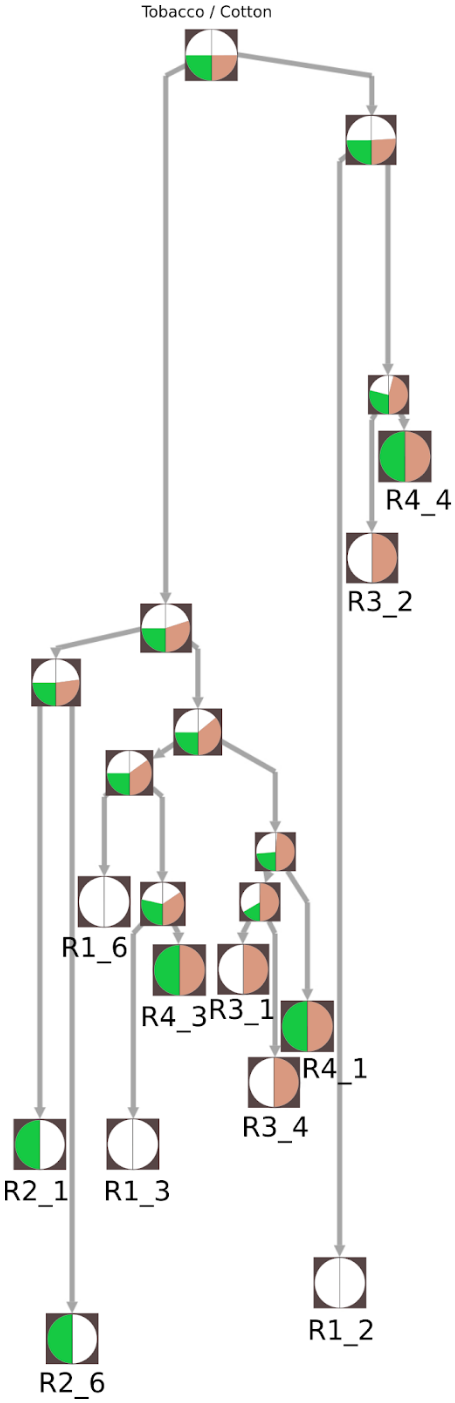

Fig. S8. Reconstruction of the ancestral states using PastML with the ML MPAA method.

M. incognita host races (R1-R4) are defined by differential compatibilities on Tobacco and Cotton. The left semicircle displays the probability of node being able to parasitise tobacco while the right semicircle displays the probability of the node being able to parasitise cotton.

Species	Primer	Sequence (5'→3')	Length (bp)	Reference
M. exigua	ex-D15F	CATCCGTGCTGTAGCTGCGAG	562	Randig et al., 2002
	ex-D15R	CTCCGTGGGAAGAAAGACTG		
M. incognita	inc-K15F	GGGATGTGTAAATGCTCCTG	399	Randig et al., 2002
	inc-K15R	CCCGCTACACCCTCAACTTC		
M. paranaensis	par-C09F	GCCCGACTCCATTTGACGGA	208	Randig et al., 2002
	par-C09R	CCGTCCAGATCCATCGAAGTC		
M. javanica	Fjav	GGTGCGCGATTGAACTGAGC	670	Zijlstra et al., 2000
	Rjav	CAGGCCCTTCAGTGGAACTATAC		

Table S6. List of the SCAR molecular markers used for *Meloidogyne* spp. confirmation
